# Mismatches between breeding phenology and resource abundance of resident alpine ptarmigan negatively affect chick survival

**DOI:** 10.1002/ece3.5290

**Published:** 2019-05-26

**Authors:** Gregory T. Wann, Cameron L. Aldridge, Amy E. Seglund, Sara J. Oyler‐McCance, Boris C. Kondratieff, Clait E. Braun

**Affiliations:** ^1^ Department of Ecosystem Science and Sustainability Colorado State University Fort Collins Colorado; ^2^ Colorado Parks and Wildlife Montrose Colorado; ^3^ Fort Collins Science Center U.S. Geological Survey Fort Collins Colorado; ^4^ Department of Bioagricultural Sciences and Pest Management Colorado State University Fort Collins Colorado; ^5^ Grouse Inc. Tucson Arizona

**Keywords:** alpine, Lagopus, NDVI, phenological mismatch, reproduction

## Abstract

1. Phenological mismatches—defined here as the difference in reproductive timing of an individual relative to the availability of its food resources—occur in many avian species. Mistiming breeding activities in environments with constrained breeding windows may have severe fitness costs due to reduced opportunities for repeated breeding attempts. Therefore, species occurring in alpine environments may be particularly vulnerable.
2. We studied fitness consequences of timing of breeding in an alpine‐endemic species, the white‐tailed ptarmigan (*Lagopus leucura*), to investigate its influence on chick survival. We estimated phenological mismatch by measuring plant and arthropods used by ptarmigan in relation to their timing of breeding.
3. We monitored 120 nests and 67 broods over a three‐year period (2013–2015) at three alpine study sites in the Rocky Mountains of Colorado. During this same period, we actively monitored food resource abundance in brood‐use areas to develop year and site‐specific resource phenology curves. We developed several mismatch indices from these curves that were then fit as covariates in mark‐recapture chick survival models.
4. A correlation analysis between seasonal changes in arthropod and food plant abundance indicated that a normalized difference vegetation index (NDVI) was likely the best predictor for food available to hens and chicks. A survival model that included an interaction between NDVI mismatch and chick age received strong support and indicated young chicks were more susceptible to mismatch than older chicks.
5. We provide evidence that individual females of a resident alpine species can be negatively affected by phenological mismatch. Our study focused on individual females and did not examine if phenological mismatch was present at the population level. Future work in animal populations occurring in mountain systems focusing on a combination of both individual‐ and population‐level metrics of mismatch will be beneficial.

## 1 INTRODUCTION

The timing of reproductive events is a critical component of fitness across a wide range of plant and animal taxa (Visser & Both, 2005). Climate change has been a major driver of alterations in the timing of reproductive events in recent decades (Parmesan & Yohe, 2003; Walther et al., 2002), particularly for birds, where most documented cases have shown earlier breeding associated with warming spring temperatures (Crick, 2004; Crick & Sparks, 1999; Dunn & Møller, 2014). The ability to time reproductive events to coincide with abundance of food resources is crucial for meeting energetic demands of both young and adults in animal populations breeding in seasonal environments (Perrins, 1970; Thomas, 2001). When the rate of the phenological response to environmental cues differs between individuals and their food resources, phenological mismatches can occur (Edwards & Richardson, 2004). Findings from studies investigating phenological mismatches between predators and prey have shown decoupled interactions can be severe for consumers (Jones & Cresswell, 2010), but loss of common interactions can be complex and may actually lead to new resource opportunities, such as exposure to previously unavailable types of food (Miller‐Rushing, Hoye, Inouye, & Post, 2010).

Phenological mismatches have been observed in long‐term studies of wild bird populations. Some examples include mistiming of migratory events between wintering and breeding areas in pied flycatchers (*Ficedula hypoleuca*) leading to birds arriving after the seasonal pulse in invertebrate food and subsequent reproductive declines (Both et al., 2006; Both & Visser, 2001), and common cuckoos (*Cuculus canorus*) arriving later to breeding areas than the hosts whose nests they parasitize (Saino et al., 2009). Indirect evidence based on mistiming between arrival dates of migratory birds and temperature variables associated with plant phenology suggests phenological mismatches in migratory species may be common (Saino et al., 2010). In some populations, this may be due to greater potential for asynchrony in weather and climate patterns as distance between wintering and breeding areas increases, which can ultimately lead to weather cues at wintering areas being misrepresentative of conditions at breeding areas. However, asynchrony in local climate regimes can also produce phenological mismatches. This mechanism can occur, for example, when a given area experiences different trends in temperature during different parts of the year (Senner, Stager, & Cheviron, 2018). Both resident and migratory species can be susceptible to such an asynchronous mechanism, but it may be more common in migratory species due to their use of many different geographic areas throughout the year compared to resident species, and therefore greater potential exists for exposure to climate regimes that are asynchronous (Senner et al., 2018).

Other important factors that are less frequently considered in phenological studies are the types of ecological systems inhabited. This is an important consideration because length of growing seasons can vary widely between ecosystems, and breeding season length can affect both the life history characteristics of populations (Bears, Martin, & White, 2009; Camfield, Pearson, & Martin, 2010; Wilson & Martin, 2011) as well as the number of breeding attempts that can be made within a season (Martin & Wiebe, 2004).

High‐elevation ecosystems are one of the most extreme examples of seasonal environments, with long winters and short growing seasons (Seastedt, 2001). Depending on the amount of snowpack and spring temperature, the start of the growing season may vary considerably from year to year. This extreme seasonality and shortened period of resource productivity suggest animals in these habitats may incur higher fitness costs if they breed too early or too late relative to the pulse in food availability (Martin & Wiebe, 2004). This prediction stems directly from limitations in the number of breeding attempts that can occur over a short growing season (Martin & Wiebe, 2004). Moreover, the frequency of extreme weather events increases with elevation, posing additional challenges for species in these environments (Martin et al., 2017). Fecundity (i.e., the number of young produced per female) in high‐elevation ecosystems tends to be lower compared to lower‐elevation ecosystems (Badyaev & Ghalambor, 2001), and fewer breeding opportunities coupled with a highly stochastic environment may greatly influence annual variability in this vital rate (Martin et al., 2017). Therefore, it is important to consider the distribution of resources throughout the growing season to better understand seasonal limitations faced by breeding species, in addition to the aforementioned abiotic factors.

A major limitation of assessing phenological mismatches in wildlife populations is the lack of appropriate data available at different trophic levels across which individual organisms may interact. For example, many long‐term studies of animal populations document changes in the timing of breeding or other behavioral changes to warming temperatures, but understanding the fitness consequences of these observed changes may not be possible to assess without corresponding information on availability of their food resources (Visser & Both, 2005). It is important to consider phenological measures of both the focal species and their primary resources to understand how much a species should be changing its reproductive phenology to track its environment (Visser & Both, 2005). This approach also provides a framework to estimate effects of phenological mismatch on individual reproduction, a key determinant of population growth.

We examined individual fitness in an alpine specialist and how it relates to environmental conditions and availability of food resources. The white‐tailed ptarmigan (*Lagopus leucura*) is the smallest species in the grouse subfamily Tetraoninae (i.e., tetraonids) and a resident endemic to alpine and subalpine habitats throughout western North America (Martin, Robb, Wilson, & Braun, 2015). Hens typically initiate nesting in the first half of June with clutch sizes varying from 2 to 8 eggs (Martin et al., 2015). Hens may renest if they lose a nest during the laying or early incubation period but will only raise one brood in a season. Two populations studied in Colorado since the late 1960s have advanced their nesting phenology significantly in response to warming springs, but the rate of change varied between populations (Wann, Aldridge, & Braun, 2016). Reproduction over this same time period significantly declined in the population that advanced its average breeding phenology the most but remained unchanged in the other (Wann et al., 2016). However, mechanisms underlying these differences were not investigated.

**Figure 1:**
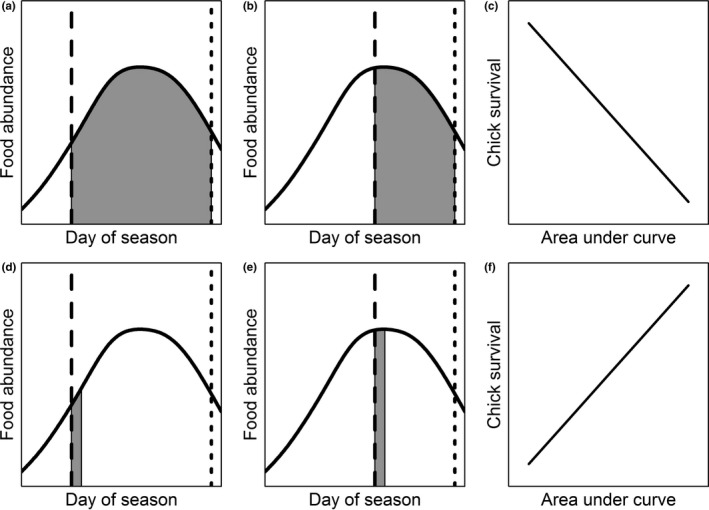
Predicted relationship between survival of white‐tailed ptarmigan (*Lagopus leucura*) chicks relative to timing of resource availability. The timing of hatch (large dashed line) is predicted to influence daily survival based on the availability of resources (solid curve). The end of the growing season is represented with small dashed line. Under the first hypothesis (first row panels), resource availability over the entire posthatch period affects chick survival, because larger areas represent missed resource opportunities (a), while small areas represent optimal timing (b), and the area of the shaded region is predicted to negatively relate to nest survival (c). Under the second hypothesis (second row panels), resource availability immediately after hatching affects chick survival, and ptarmigan young may hatch at a time that does not coincide with peak resource abundance (d) or does coincide with peak resource abundance (e), in which case the area of the shaded region is predicted to positively relate to nest survival (f). Note that the resource availability curve can represent any resource (e.g., plant biomass or insect biomass). In the case of plant productivity, almost all broods monitored hatched before or within five days of the peak

We initiated a three‐year telemetry study to investigate potential drivers of reproductive rates from three populations, two of which were long‐term study sites. We tracked individual hens throughout the breeding season to collect information on chick survival, in addition to temporal changes in alpine plant and insect abundance. Other environmental factors were also considered, including weather events during the brood‐rearing periods, which are known drivers of reproduction in birds (Martin et al., 2017). Our objectives were twofold. First, we investigated relationships between primary plant productivity and food availability at our study sites. We asked the question, how does plant productivity relate to phenology of forage forbs and insect prey? We measured temporal changes in plant productivity (as measured by a normalized difference vegetation index [NDVI]) at all our study sites, and insect abundance at a subset of years and sites, and predicted the seasonal relationships using generalized additive models. We examined consistencies of patterns across years and sites and used cross‐correlation functions to assess correlations between known forage forbs and plant productivity, and insects and plant productivity. Second, we tested whether chick survival in ptarmigan varied as a function of phenological differences between timing of reproductive events relative to plant resources. We asked the question, do phenological mismatches lead to decreased daily chick survival rates? Unlike many previous phenological studies that used only simple metrics such as a date mismatch (the difference in days between date of resource peak and date of median hatch), we additionally used estimates of changing temporal abundance for plant productivity and forage forbs to calculate indices of mismatch (Figure 1) to address this question. These indices of phenological mismatch were then fit as individual‐level covariates to capture–recapture models to predict daily chick survival.

## 2 MATERIALS AND METHODS

### 2.1 Study area

**Figure 2:**
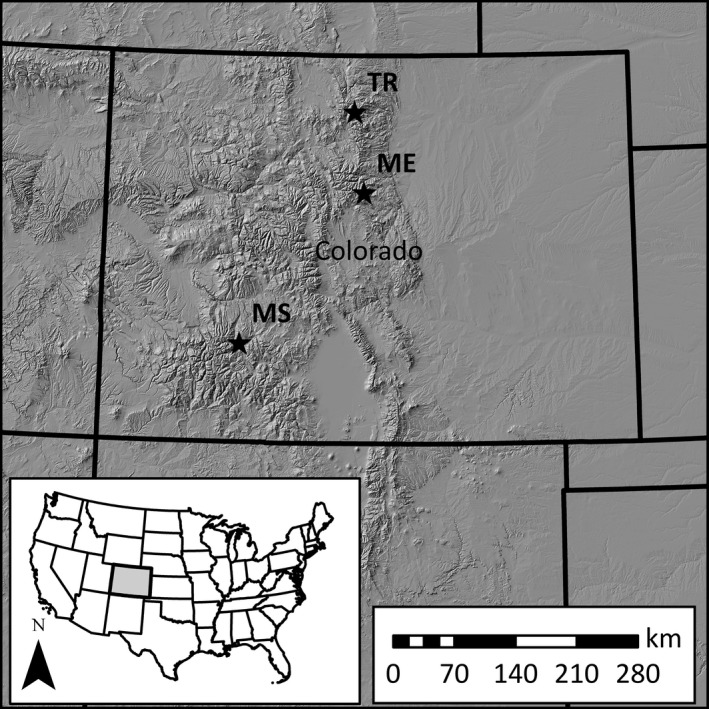
Locations of sites where white‐tailed ptarmigan (*Lagopus leucura*) were studied in Colorado, USA from 2013 to 2015. Study sites were at Mt. Evans (ME), Mesa Seco (MS), and Trail Ridge (TR)

Data were collected from 2013 to 2015 at three alpine sites in the southern Rocky Mountains (Figure 2). The Mt. Evans (ME, 39°35′N, 105°37′W) and Trail Ridge (TR, 40°25′N, 105°45′W) sites were in the Front Range in north central Colorado, and the Mesa Seco (MS, 38°1′N, 107°14′W) site was in the San Juan Mountains in southwestern Colorado. The study area extents and elevational ranges [ER] were 7.0 km^2^ (ER: 3,535–4,270 m) at ME, 9.1 km^2^ (ER: 3,505–3,688 m) at TR, and 3.3 km^2^ (ER: 3,718–3,900 m) at MS. Study areas were managed by the U.S. Forest Service (ME and MS) and U.S. National Park Service (TR). Weather was highly seasonal at all sites with precipitation falling in the form of snow throughout the nonbreeding season (September–April) and rain or hail during the breeding season (May–August). Average monthly temperatures for all sites were warmest in July (7.9°C) and coldest in December (−9.4°C). Elk (*Cervus canadensis*) and domestic sheep (*Ovis aries*) grazing were both common at our TR and MS sites (respectively). Vegetation was typical of alpine habitats in Colorado and has been previously described (Braun, 1969). Briefly, they consisted primarily of willow (*Salix* spp.) and Engleman spruce (*Picea engelmannii*) communities at lower elevations, and higher‐elevation communities were dominated by grasses (e.g., *Deschampsia cespitosa* and *Poa* spp.), sedges (*Carex* spp.), and forbs (e.g., *Geum rossii*, *Trifolium* spp.).

### 2.2 Reproductive data

Field protocols were approved by the Colorado State University Institutional Animal Care and Use Committee (IACUC, protocol # 12‐3352A). Female ptarmigan were located in May and early June by locating males paired with hens using broadcasts of male territorial calls (Braun, Schmidt, & Rogers, 1973) or by scanning the edges of snowfields with binoculars. Using a modified noose (Zwickel & Bendell, 1967), we captured hens. A 9‐g radio transmitter with an elastic collar was fit to each hen (Model RI‐2D, Holohil, Ltd., Carp, Ontario), in addition to an aluminum State of Colorado band and 2–4 plastic colored bandettes for individual identification. Nest contents were inspected on the 10th day a nest was known to exist by nudging hens off the nest and counting the eggs. Hens with successfully hatched nests were visually located the first day they were no longer observed on a nest using binoculars from a distance of 5–20 m to count the number of chicks. Hens were located 2–3 times weekly from spring until the second week of September.

### 2.3 Resource sampling and phenology

Brood‐use areas were identified at two of three study sites (ME and TR) prior to the start of the study based on 43 years of long‐term reproductive data (Wann et al., 2016). Brood locations recorded from 1966 to 2012 were used to delineate areas that were the focus of vegetation sampling. We had limited prior knowledge of brood‐rearing locations at MS based on brood observations obtained during surveys in 2012. Locations from these surveys were used to delineate brood‐use areas in the same manner as ME and TR, but we updated the delineated areas following the 2013 season due to the addition of newly observed use locations. Detailed methodologies for delineation of brood‐use areas and generation of sample points are outlined in Appendix S1: SM 1.

Plant phenology and productivity within brood‐use areas were monitored throughout the breeding season to estimate site‐specific variation. This was done by generating random sample points within brood‐use polygons which we visited at weekly intervals. Each sampling point consisted of a 1‐m^2^ area marked with two wood stakes placed at opposite corners of the sampling quadrat. Information recorded at each sampling period included the genus or species of plant in bloom (i.e., presence of flower or seed head), time of day, and a NDVI photograph (Figure S1). NDVI photographs were taken with a standard digital camera modified to record both visible and near infrared light (Canon ELPH 110, MaxMax, Ltd., Carlstadt, NJ). Photographs were processed using scripts written in the ImageJ language (Schneider, Rasband, & Eliceiri, 2012) by extracting pixel‐specific RGB (red–green–blue) values and calculating an average NDVI value for each image (Figure S2). Detailed methods for analysis of NDVI photographs and timing of bloom are described in Appendix S1: SM 2.

Temporal changes in invertebrate abundance were measured by sampling 20‐m insect transects within the delineated brood‐use areas at ME (2013–2015) and MS (2013–2014). Invertebrate abundance could not be measured at TR due to sampling restrictions within the park. Sticky aphid papers (Seabright Laboratories) were pinned to the ground along transects (locations in Table S1). We collected and replaced aphid papers every seven days and identified individual invertebrates to the lowest taxonomic level which was either family or genus for the majority of samples. Counts of invertebrates were used to estimate a weekly transect density for each specific taxonomic category, and density was calculated for each taxon and year for each sample paper, transect, and week, by dividing the sum of the total count by the sum of paper area. Additional details on invertebrate sampling and calculation of abundance are presented in Appendix S1: SM 3.

Generalized additive models (GAMs) were used to predict daily changes in plant bloom, plant NDVI, and invertebrate abundance. We fit GAMs to our data using the mgcv package (Wood, 2006) in R (R Core Team, 2013). Models were fit using either the density (insects) per transect or average NDVI (plant productivity) per point as the response variable and Julian day of the observation as a covariate. A third type of GAM was also fit to plant data which used the presence (coded as 1) or absence (coded as 0) of bloom or seed head as a response variable, Julian day as a covariate, and a binomial link function. We calculated cross‐correlation coefficients in R to assess the phenological relationships between predictions for forage forbs, insects, and plant productivity (measured by NDVI). Cross‐correlations provided a way to calculate correlations between two time series at different daily time lags which provided us with information on the number of days before or after the peak in plant productivity resources at which the correlations were highest (methods described in Appendix S1: SM 4).

### 2.4 Chick survival

We estimated daily chick survival using open‐population survival models, which allowed for counts of young within family groups (Lukacs, Dreitz, Knopf, & Burnham, 2004). The young‐survival model is an extension of the Cormack–Jolly–Seber model (Williams, Nichols, & Conroy, 2002) and consists of parameters for apparent survival (*ϕ*) and recapture probability (*p*). Data on the number of young with attending and individually marked adults are used to construct individual encounter histories consisting of counts of chicks observed during each encounter period. Survival in the model refers to survival of individual chicks within broods. Encounter histories for individual broods were standardized so the first encounter occasion represented the date of hatch for all broods. For each brood, the first entered value in the encounter history was based on the number of hatched eggs documented at the nest. We constructed encounter histories for each hen over a 42‐day period posthatch. All nest and chick survival models were fit in program MARK (White & Burnham, 1999) using a logit link function. One assumption of the young‐survival model is that attending adults do not adopt young. Chick adoptions probably occurred at low rates at our sites (~1% of encounter occasions), and we examine the potential effects on model selection in Appendix S1: SM 5.

### 2.5 Covariates and model selection

We used the Akaike's Information Criterion adjusted for small sample sizes (AICc) to choose the best model in a candidate model set (Burnham & Anderson, 2002) based on the lowest AICc value. A hierarchical approach (two‐step) was used to pick the base structures for our models, followed by fitting covariates. Determining the best base structure consisted of constructing five structures for parameter *ϕ* and *p* with different additive and interactive combinations for year, site, and chick age effects. These models included a simple structure with only year or site effects, an additive relationship between year and site (year + site), and an interaction between year and site (year × site). To choose the best structure for parameter *p*, the parameter *ϕ* was kept in the most general form (site × year) and the five different structures of *p* were then compared using AICc. The structure of *p* from the model with the lowest AICc was then used to compare the five structures for *ϕ*, and the model receiving the lowest AICc was included in the candidate model set. Also, prior to construction of candidate models for daily chick survival, we examined if chick age was an important predictor. Previous studies found mortality is highest for grouse chicks during the first few weeks of life, and survival increases as chicks age (reviewed by Hannon & Martin, 2006). We found that increasing age through the first 18 days posthatch followed by constant survival was a strong predictor of daily chick survival (covariate termed CAGE). We included this effect both by itself and as an additive and interactive component with the best base structure for survival. Again, the structure receiving the lowest AICc value was used to fit covariates (described below).

The second stage of building the candidate model set considered several covariates to explain variation in chick survival. We added covariates to our base model structure chosen during the first stage of model selection. Additional modeling of parameter *p* was not considered because environmental covariates were not thought to affect our ability to detect chicks as brood detection was through the hen. Weather variables were fit as individual covariates. Weather covariates for cumulative precipitation (precip) and the sum of minimum (min) and maximum (max) temperatures were calculated over an 18‐day period posthatch. We calculated the time difference [TDM] between peak plant productivity and date of hatch (TDM = date of hatch − date of peak NDVI) to estimate the effects of timing of breeding relative to availability of food resources. We estimated resource abundance more directly by calculating the area under the curve predicted from the GAM model between hatch date and 28 August, a date after which vegetation on all our plots had typically begun to senesce, as a measure of seasonal mismatch. These mismatch indices were calculated for the area under the NDVI‐derived productivity curve (seasonal mismatch [SeasM]), area under the predicted timing of bloom curve (i.e., area mismatch calculated for four forage genera: *Geum* [GeumM], *Trifolium* [TrifM], *Artemisia* [ArteM], and *Polygonum* [PolyM]), and summed area under all the curves of bloom species (ForbM). We chose forage forbs for species we knew were consumed by ptarmigan (May & Braun, 1972). Increasing values of the seasonal covariates were predicted to correlate with declines in daily chick survival because higher values indicate broods are reared at a time that does not coincide with peak resource abundance (Figure 1, top row). We also tested a covariate for NDVI the day a nest hatched (PostM) and predicted the relationship would be positive, because high resource availability at this time would be immediately beneficial for chicks (Figure 1, bottom row). Each covariate was considered individually as additive effects with the base structure chosen for *ϕ* and as interactive effects with chick age (e.g., TDM × CAGE). We did not construct models for all possible subsets of covariates because doing so would have resulted in an extremely large candidate model set.

Insects were only measured at two sites for a total of five years, and they could not be directly used as covariates in survival models. We instead made inferences from the correlation analysis previously described.

## 3 RESULTS

### 3.1 Arthropod and plant phenology

We captured many different arthropod taxa (Table S2), the most abundant of which were members of families Muscidae (flies, mostly species of the genus *Thricops*) and Acrididae (grasshoppers, mostly *Aeropedellus clavatus*), and several butterfly families, including Papilionidae (*Parnassius smintheus*), Nymphalidae (mostly *Boloria* spp.), and Pieridae (*Colias meadii*). Grasshopper abundance increased continuously across the breeding season for all years, even after plants began to senesce, at both ME (Figure S3) and MS (Figure S4), and correlation coefficients suggested plant productivity preceded grasshoppers at ME (Figure S5) but not at MS (Figure S6). Fly abundance generally peaked prior to the peak in plant productivity at ME (Figure S3), but showed no clear pattern at MS (Figure S4) across years, which was further demonstrated by their associated correlation coefficients at both sites (Figures S5 and S6). Papilionidae and Pieridae consistently preceded plant productivity at ME (Figure S3), but did not lag plant productivity at MS (Figure S4), which was consistent with the correlation coefficients for these families at both sites (Figures S7 and S8, respectively). A summary of correlation coefficients for arthropods is available in Table S3.

**Figure 3:**
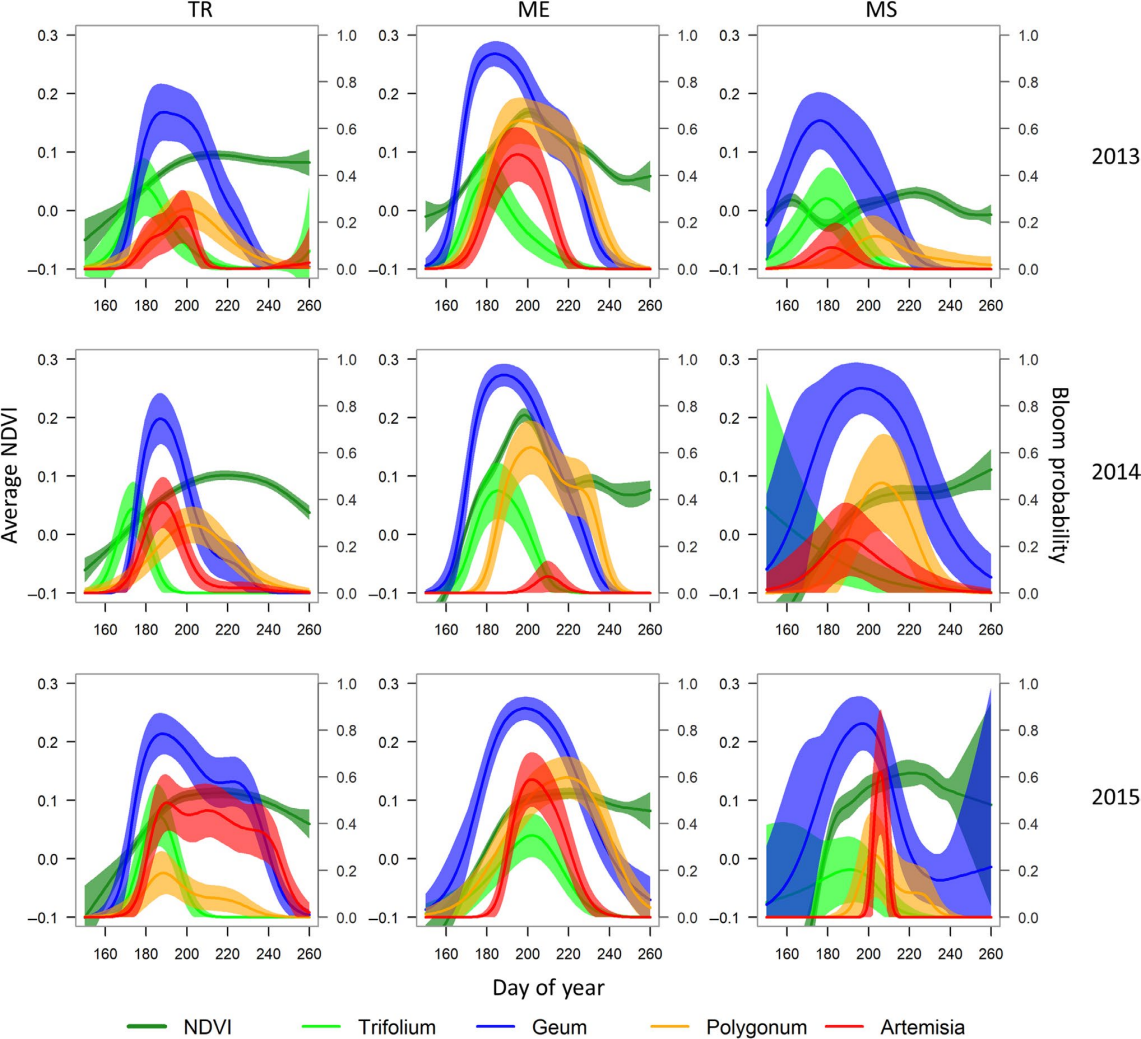
Estimated phenological relationships of plants as a function of day of year at three alpine sites in Colorado, USA. Average NDVI (left *y*‐axis) and bloom probability of four different forb species (right *y*‐axis) were predicted for each day of the season using a generalized additive model fit to data collected at 1‐m^2^ plots surveyed at a weekly interval. Shading represents 95% confidence intervals of predictions. Study sites were at Trail Ridge (TR), Mt. Evans (ME), and Mesa Seco (MS)

We collected 3,704 NDVI photographs during the breeding season from 2013 to 2015 at 126 sampling points. Timing of peak maximum NDVI (estimated with GAMs and averaged across sampling points) varied by site and year, with ME having the earliest average peak NDVI (26 July) followed by TR (5 August) and MS (16 August) (Figure 3). Comparisons between NDVI and forage species’ bloom suggested peak NDVI generally lagged forage bloom, and these patterns were consistent across sites and years (Table S4). Species in the genus *Trifolium* (*T. nanum* and *parryi*) were the earliest bloomers at our study sites, followed by *Geum rossii*, and species in the genus *Artemisia* (*A. frigida* and *A. scopulorum*) and *Polygonum* (*P. viviparum* and *bistortoides*). Species of *Polygonum*, which are highly important forage forbs for ptarmigan, closely coincided with peaks in plant productivity with average time lags of −2.6 days. Overall, the correlation relationships were strongly consistent within sites, and generally consistent among years (Figures S9–S11). A summary of correlation coefficients for forage forbs is available in Table S4.

### 3.2 Chick survival

Eighty‐one hens produced 120 nests during the breeding seasons from 2013 to 2015 (Table 1). Twenty‐nine of 81 hens contributed >1 nests to the sample because they were monitored in multiple years or renested. Twelve nests resulted from renesting. Of all nests inspected, adults laid larger average clutches ($\bar{x}$ = 5.7 eggs, range = 3–7 eggs, *n* = 61 nests) than subadults ($\bar{x}$ = 5.2 eggs, range = 3–7 eggs, *n* = 31 nests). No evidence of nest abandonment was found at any of our sites during the study. A total of 67 nests successfully hatched one or more eggs (56% nest success). Nest inspections were conducted at 61 of 67 successful nests for a total count of 330 eggs, 301 of which hatched (91% egg viability).

**Table 1:** White‐tailed ptarmigan (*Lagopus leucura*) nests and broods monitored in Colorado from 2013 to 2015

Description	Year			Grand total
	2013	2014	2015	
Trail Ridge (TR)				
Nests total	10 (10)	15 (14)	12 (11)	37 (26)
Successful nests	6 (6)	8 (8)	8 (8)	22 (20)
Successful broods	6 (6)	5 (8)	4 (4)	15 (15)
Mt. Evans (ME)				
Nests total	13 (13)	21 (18)	22 (19)	56 (37)
Successful nests	7 (7)	11 (11)	8 (8)	26 (23)
Successful broods	4 (4)	5 (5)	4 (4)	13 (12)
Mesa Seco (MS)				
Nests total	9 (8)	8 (8)	10 (10)	27 (18)
Successful nests	7 (7)	7 (7)	5 (5)	19 (13)
Successful broods	2 (2)	5 (5)	2 (2)	9 (9)
Grand total (all sites)				
Nests grand total	32 (31)	44 (40)	44 (40)	120 (81)
Successful nests grand total	20 (20)	26 (26)	21 (21)	67 (56)
Broods grand total	20 (20)	26 (26)	21 (21)	67 (56)
Successful broods grand total	12 (12)	15 (15)	10 (10)	37 (36)

Note

The number of females contributing to samples is provided in parentheses. Note that some females were monitored in multiple years, which is reflected in the total column and rows. Broods were monitored from each successful nest. Data were collected at Mt. Evans (ME), Mesa Seco (MS), and Trail Ridge (TR) in Colorado, USA.

We monitored 327 chicks from 67 broods from 2013 to 2015 at our study sites (Table 1). Chicks used in the analysis originated from 57 hens and young were attended by adults in 61% of broods (43/67) and subadults in 39% of broods (24/67). The average brood size at hatch for both age classes combined was 4.9 chicks, with adults averaging the largest broods ($\bar{x}$ = 5.1 chicks, range = 1–7 chicks, *n* = 43 broods) followed by subadults ($\bar{x}$ = 4.4 chicks, range = 1–6 chicks, *n* = 24 broods).

We evaluated 23 chick survival models in our candidate model set (full model set Table S5). The first stage of model selection indicated recapture probability (*p*) varied by site, but not year. Estimates for *p* were high (ME = 0.912, 95% CI = 0.890–0.930; MS = 0.953, 95% CI = 0.915–0.974; TR = 0.881, 95% CI = 0.851–0.906), indicating chicks were highly detectable during brood visits if they were alive and with the brood hen. Estimates of daily survival (*ϕ*) varied by site (ME = 0.968, 95% CI = 0.960–0.974; MS = 0.949, 95% CI: 0.933–0.960; TR = 0.978, 95% CI: 0.971–0.983). Chick age was an important component of the base model structure. The best base structure for daily survival (*ϕ*) included an interaction between site and year, and an interaction between chick age and site (i.e., *ϕ*{*b*0 + site + year + CAGE + site × year + site × CAGE}), supporting daily chick survival variation by site, year, and age. This was the base structure used in all covariate models.

**Figure 4:**
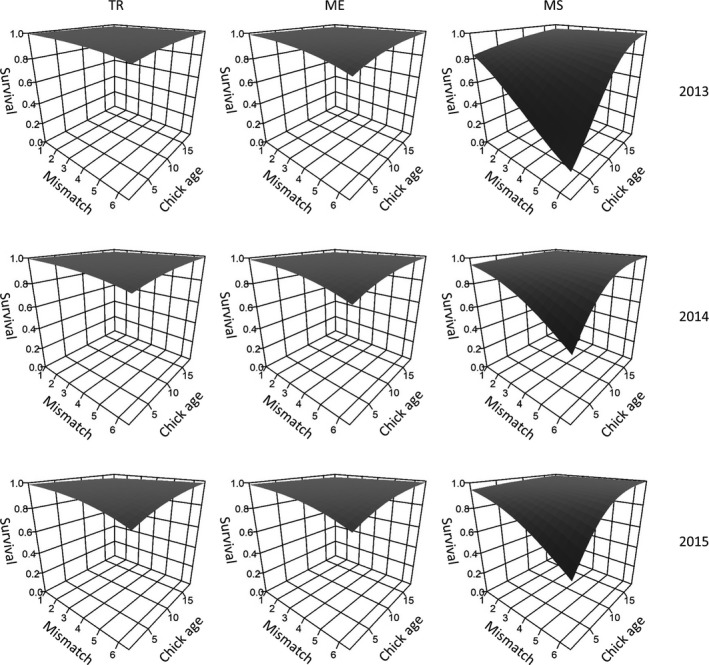
Apparent survival estimates of white‐tailed ptarmigan (*Lagopus leucura*) chicks studied at three populations in Colorado, USA. Predictions of apparent survival are taken from the highest supported model and plotted as a function of chick age and phenological mismatch for each site and year combination. Study sites were at Trail Ridge (TR), Mt. Evans (ME), and Mesa Seco (MS). Predictions were produced from the top‐ranked chick survival model

Generally, covariates for weather and plant productivity metrics improved model likelihood over the best base structure. A model with an interaction between chick age and plant seasonal mismatch (i.e., *ϕ*{*b*0 + site + year + CAGE + site × year + site × CAGE + SeasM + CAGE × SeasM}) received 93% of model support and predicted chicks less than 19 days of age to have lowest chick survival when seasonal mismatch of plant productivity (SeasM) was high, but the effect was additive to the effects of year and site (Figure 4). In contrast, the model with an interaction between chick age and NDVI the day a nest hatched (PostM) received no support. The average percent change of daily apparent chick survival under mismatch values of 1 to values of 6 was 15.1%, but the percent changes varied considerably among sites and years, with a lowest change of −3.7% at TR in 2013, and a highest change of −47.1% at MS in 2013 (Table S6).

## 4 DISCUSSION

Timing of reproductive events can be a critical component of fitness for animals living in seasonal environments (Perrins, 1970; Thomas, 2001). Our own study focused on the fitness consequences of phenological mismatch in individual female ptarmigan that are resident within alpine and subalpine ecosystems. We were interested in investigating the potential consequences of variation in reproductive phenology in a mountain system because the window of resource availability in these environments is much narrower than those at lower elevations. Therefore, the mistiming of breeding activities could have important implications for mountain species if its occurrence is frequent enough to be measurable at the population level. Our study was limited to investigating individual variation in one reproductive rate, chick survival, of female ptarmigan. Much additional work on the influence of reproductive phenology on animal populations in mountain ecosystems remains given that individual‐based studies are critical to investigate mechanisms that may underlie population‐level responses (Clutton‐Brock & Sheldon, 2010). In the discussion that follows, we begin with chick survival and its relationship to the resource phenology curves we estimated. We then discuss previous long‐term work at two of the populations we studied and how it relates to phenological studies of other tetraonids to provide additional context for our findings.

### 4.1 Drivers of chick survival

To test whether fitness consequences existed for females raising chicks during times that were out of synchrony with food resources, we used the areas under the estimated resource phenology curves available to chicks. In this context, we made two different predictions (not mutually exclusive) on resource availability and chick survival. First, we calculated a seasonal mismatch index that considered resource availability over the seasonal period following hatch (represented by the SeasM covariate). Second, we calculated a posthatch mismatch index that considered resources available immediately following hatch (represented by the PostM covariate). Our top model received strong support and predicted that daily chick survival was lower for chicks that hatched at high values of seasonal mismatch for NDVI. In contrast, the posthatch mismatch index for NDVI was not well supported, indicating that variability in resource abundance immediately after hatch did not strongly affect chick survival. This result may seem surprising, but in the case of tetraonid chicks, nourishment is obtained primarily from an invaginated yolk sac the first few days after hatching (Bergerud, 1970; Marcström, 1960), suggesting that perhaps longer‐term conditions of food availability are likely to be more important. Longer‐term conditions were better captured by the seasonal mismatch index. Furthermore, the most basic measure of phenological mismatch we considered was the difference between day of hatch and day of peak NDVI (i.e., time mismatch, represented by the TDM covariate), but models containing this covariate did not receive support. This potentially demonstrates one benefit of considering the area under the phenology curve, which captures additional information on resource availability during the crucial growth period for chicks. The utility of phenology curves for phenological mismatch studies has been recognized by many ecologists (Miller‐Rushing et al., 2010; Visser & Both, 2005). However, most studies do not use the area under the phenology curve as a covariate as we did (but see Vatka, Orell, & Rytkönen, 2016). Instead, they are typically used to identify the day of year at which resources peak. Population statistics for the differences between a measure, such as median peak hatch date and peak NDVI, are then taken as a covariate to fit in models explaining population‐level measures of reproduction (Ross, Alisauskas, Douglas, & Kellett, 2017), such as the mean number of fledged chicks per female. This approach is likely to capture meaningful information about the optimal timing of breeding relative to food resources. However, it is also true that the phenology curves themselves provide additional information that can be utilized (Vatka et al., 2016), including the overall abundance of resources in one year relative to another.

We did not directly fit mismatch indices derived from the estimated insect phenology curves as covariates in our chick survival models. This was due to an incomplete sample of insect data for each site and year. Instead, our inference was based on cross‐correlations between plant resource phenology curves (NDVI) and insect resource phenology curves for sites and years where data existed for both to understand their relationships. Previous work has demonstrated the importance of insects as a food source for tetraonid chicks during the first few weeks posthatching. For example, May (1975) identified crop contents of white‐tailed ptarmigan chicks collected at <3 weeks of age and found that various invertebrates comprised up to 63% of dry weight. Lepidoptera larvae formed a significant quantity of the invertebrates consumed, consistent with other tetraonid species, including black grouse (Baines, Wilson, & Beeley, 1996; Wegge & Kastdalen, 2008), willow ptarmigan (Spidso, 1980), and capercaillie (Picozzi, Moss, & Kortland, 1999). We found the highest cross‐correlations between NDVI and three families in Lepidoptera. We did not capture sufficient quantities of Lepidoptera larvae to estimate temporal changes in their abundance. However, our traps captured sufficient numbers of adults to estimate temporal changes in abundance, which we presume should be characteristic of changes in larval abundance, albeit with a lag effect (i.e., the number of days between the larvae stage and maturation to an adult class). Overall, cross‐correlations were consistent between years, indicating NDVI may provide a useful index for temporal trends in Lepidoptera taxa at alpine sites. Nevertheless, we acknowledge that inference from our results with respect to insect availability during the posthatch period requires the assumption that NDVI is representative of a subset of insects such as Lepidoptera and their consistent relationships with NDVI between sites and years. Since Lepidoptera larvae were not identified to species by May (1975), it is not currently known if ptarmigan chicks at our sites specialize on any particular species. Future work would benefit from a more intensive insect sampling design and the inclusion of pitfall traps to directly capture Lepidoptera larvae. In contrast to insects and NDVI, the relationship between NDVI and food plants produced extremely consistent patterns for nearly all taxa comparisons, indicating that NDVI phenology curves contain a large amount of information about temporal changes in the abundance of plants consumed by ptarmigan. Therefore, much less uncertainty exists in the value of NDVI as an index for food plants, and temporal estimates of NDVI are likely to be the best overall representation of changing food availability at our alpine sites.

Weather effects were included in several of our candidate models, including temperature and precipitation measures that were summed over an 18‐day posthatch period for each brood. The second‐best ranked model included a precipitation effect, although it received a modest amount of support relative to the top model, and the effect was contrary to expectations (daily chick survival was positively related to cumulative precipitation). Nonetheless, several models containing weather covariates still offered an improvement over the base model structure. Cold and wet conditions have been demonstrated to negatively influence willow ptarmigan (*Lagopus lagopus*; Steen et al., 1988) and rock ptarmigan (*Lagopus muta*; Novoa, Besnard, Brenot, & Ellison, 2008) chicks, possibly through lost feeding opportunities (Jorgensen & Blix, 1985; Pedersen & Steen, 1979). Therefore, we are reluctant to suggest weather is unimportant for ptarmigan chick survival in the populations we studied simply because they were not in our top‐ranked model. Summing weather effects over an 18‐day posthatch window as we did may have been too coarse to capture extreme conditions that could contribute to chick survival. Our study may have also been too short in duration to capture weather conditions that were extreme enough to influence chick survival, which can be important for alpine birds (Martin & Wiebe, 2006; Martin et al., 2017). We suspect the latter may be likely, as we also examined effects such as number of days with >1 cm of rain (among other thresholds) in an exploratory analysis that occurred early in the modeling process, but no support was found for these models, perhaps because these events occurred infrequently enough to only occur in a very small proportion of individual covariates. Chick survival also varied considerably among our study sites, indicating site‐specific characteristics were important. Average chick survival was highest at TR and lowest at MS. We observed many predators at MS in 2013 (primarily ermines), which likely contributed to the low observed nest and chick survival rates during that year. Site‐level effects were not of direct interest in this study, and we viewed them as a nuisance that needed to be accounted for through their inclusion in our models. We believe this was a sensible approach as it was quite obvious that the effects of site, year, and chick age were responsible for the majority of variability present in chick survival (Figure 4). Nonetheless, mismatch and weather indices provided further improvements over the base model structure (i.e., those including the aforementioned effects), indicating their importance.

### 4.2 Relationship to previous studies

Our study was limited to 3 years and thus we did not test for the long‐term presence of phenological mismatch in our populations. However, our research was largely motivated by past long‐term monitoring of ptarmigan and other phenological mismatch studies of tetraonids. A previous analysis of 45 years of reproductive data collected at our ME and TR sites indicated that both populations significantly advanced their timing of hatch over these time spans, and timing of hatch was negatively related to spring temperature, which significantly warmed over the study period (Wann et al., 2016). A long‐term study of black grouse in Finland found that populations responded to warming springs by advancing egg‐laying and hatching (Ludwig et al., 2006), similar to findings from the ME and TR sites. However, unlike spring temperature, early summer temperature did not warm at a similar rate, meaning black grouse chicks were hatching earlier in colder temperatures, leading to higher chick mortality and population declines. A more recent study of black grouse and capercaillie in Finland observed a different response to changing spring temperatures. Wegge and Rolstad (2017) found that early spring temperatures advanced nearly three weeks at the populations studied, while late spring temperatures (when nests hatch) remained unchanged. Since mating phenology advanced only a few days in both species, nests hatched during periods with average temperatures that did not significantly warm or cool throughout the study. Breeding success actually increased for both species, which the authors suggested may have been due to improved prebreeding conditions for females, leaving the authors to conclude that warming springs were actually beneficial for black grouse and capercaillie over the period studied. Therefore, no asymmetrical mismatches in weather occurred. Similar to findings from Wegge and Rolstad (2017), no asymmetrical rates of changing weather between the nesting and posthatching period were found at the ME and TR sites (Wann et al., 2016). This leads us to believe that a population‐level phenological mismatch in reproductive rates resulting from asymmetrical climate patterns is probably unlikely in our populations, because the temperature and precipitation conditions under which broods are raised have so far remained unchanged. Nonetheless, we did observe that the population which increased its timing of breeding the most (TR) also experienced significant declines in the number of chicks per hen produced. Unfortunately, Wann et al. (2016) lacked explanatory data that could be used to test whether a mismatch between food availability and timing of breeding activities was present which could explain long‐term reproductive trends.

Unlike the aforementioned studies, our own research focused on phenological mismatches between ptarmigan chicks and food resources, and whether or not they can have a measurable effect on chick survival. Whereas a phenological mismatch between weather conditions such as temperature and precipitation would presumably operate by increasing mortality rates of chicks due to their susceptibility to cold and wet conditions (i.e., direct effects), a phenological mismatch with food resources would likely influence chick survival due to calorie deficits leading to malnourished chicks and low survivorship (Pedersen & Steen, 1979), as they would potentially be more susceptible to both weather and predation events when in poor condition (i.e., indirect effects). A future study using individual radio‐marks on young ptarmigan chicks could provide known‐fate data to test this assumption, although such a study would need to contend with the possibility of radio‐marks influencing chick survival (Hubbard, Garner, & Klaas, 1999; Hubbard, Tsao, Klaas, Kaiser, & Jackson, 1998; Larson, Clark, & Winterstein, 2001).

Our work demonstrates the potential for phenological mismatches to occur in an alpine‐endemic species of ptarmigan occurring in a highly seasonal environment when individual females breed at times that are asynchronous with food resources. However, it is important to note that just because phenological mismatches leading to lowered chick survival can occur in our populations does not necessarily mean they are occurring at frequencies high enough to lead to population‐level responses (e.g., changes in abundance). In fact, individual‐level responses to phenological mismatch may be strong, but not lead to strong population‐level responses (Reed, Jenouvrier, & Visser, 2013). For example, if females that breed at the optimal time period have substantially higher productivity than those that breed later, and there is little competition for resources during this period (i.e., most individuals are mistimed), losses due to mismatch could be offset in the presence of such density or frequency dependence (Reed, Grotan, Jenouvrier, Saether, & Visser, 2013; Reed, Jenouvrier, et al., 2013). Therefore, we are careful to avoid implying that susceptibility of individual female fitness to phenological mismatch necessitates that population‐level metrics such as number of young produced per female will decline. We found that the number of chicks produced per monitored female (from the nesting period through fledgling age) during our study was within the range observed from long‐term survey data at TR and ME (presented in Wann et al., 2016). Therefore, the rates of productivity observed during our years of study were consistent with typical levels that occur at our populations. An additional study would be needed to test whether phenological mismatches are prevalent enough to drive annual variability in reproductive rates, which would require additional years of data.

## 5 CONCLUSIONS

Phenological mismatch studies in high‐elevation ecosystems are interesting because of extreme seasonality and short growing seasons during which breeding attempts can occur. Our own work provides a unique approach to estimating resource phenology curves that may be useful in other phenology studies, and not only those occurring in mountainous systems. Testing multiple different metrics of mismatch may provide interesting insights. Our study demonstrated that some of the individual variation in chick survival can be explained by phenological mismatch.

## CONFLICT OF INTEREST

None declared.

## AUTHORS CONTRIBUTIONS

GTW, CLA, and SOM conceived of ideas, AES supervised collection at the MS site, BCK helped with design of arthropod sampling, and CEB provided expertise in ptarmigan biology. All authors contributed critically to the drafts and gave final approval for publication.

## Data Availability

MARK file containing chick survival models are available at Dryad (datadryad.org; https://doi.org/10.5061/dryad.m0s2k02).
