# Supplementary material for: Mismatches between breeding phenology and resource abundance of resident alpine ptarmigan negatively affect chick survival
Source: Ecol Evol. 2019 May 26;9(12):7200–12. doi: 10.1002/ece3.5290 (PMC6662402; doi:10.1002/ece3.5290)
Supplement: Supplementary file 1 [file ECE3-9-7200-s001.docx]

**Mismatches between breeding phenology and resource abundance of resident alpine ptarmigan negatively affect chick survival**

Gregory T. Wann^a*^, Cameron L. Aldridge^a^, Amy E. Seglund^b^, Sara Oyler-McCance^c^, Boris C. Kondratieff^d^, Clait E. Braun^e^

^a^Department of Ecosystem Science and Sustainability Colorado State University, Fort Collins, USA

^b^Colorado Parks and Wildlife, Montrose, USA

^c^U.S. Geological Survey, Fort Collins Science Center, Fort Collins, USA

^d^Department of Bioagricultural Sciences and Pest Management, Colorado State University, Fort Collins, USA

^e^Grouse Inc., Tucson, USA

*Corresponding author: greg.wann@gmail.com

# Supplementary Text

## SM 1: Delineating brood-use areas and generating sampling points

Brood-use locations at ME and TR were identified prior to the start of our study based on 45 years of long-term reproductive data (ME: 1968–2012; TR: 1968–2000 and 2010–2012) in the form of brood locations. Brood locations were used to delineate areas for the focus of vegetation and invertebrate sampling. Location data for broods were collected from 1 August to 15 September in two different ways during the study. The first form of location data consisted of brood locations recorded on topographic maps during the first 33 years of the study (1968–2000). This accounted for a subset of location data during this time period. The accuracy of these locations could not be evaluated directly, but the distinct topography of the study sites made identifying key features easy, and an accuracy of ± 100 m is likely a reasonable estimate of the error in these location data. Location data were subsequently converted to UTM coordinates in the NAD 1927 datum, followed by conversion to the WGS 1984 datum using the projections and transformation tool in ArcToolbox (Esri). The second form of location data consisted of brood locations recorded using a handheld GPS device (1998–2000 and 2007–2012; ME only). There was a gap in data from 2001–2006 at ME and 2001–2009 at TR where location data were not recorded. Location data from both maps and GPS were pooled to identify the areas and extent of brood use.

Brood-use areas were delineated based on UTM coordinates for brood observations creating polygons with a 100-m buffer around brood clusters using ArcMap 10.1 (Esri). Polygons were manually sketched around brood clusters to better approximate the spatial distribution of used areas and to ensure random sample points were generated in suitable habitat. Several of the delineated polygons in TR had areas where samples could not be collected due to restrictions outlined in our scientific research permits. These restrictions included avoiding sampling in high visibility areas near trails, parking areas, and overlooks (TR and ME), or bordering roads (TR only). As a result, portions of some delineated polygons were unavailable for sampling. We had limited prior knowledge of brood-rearing locations at MS based on brood locations obtained during surveys in 2012. Locations from these surveys were used to delineate brood-use areas as was done for ME and TR, but we revised the delineated sampling areas following the 2013 season due to the addition of newly observed use locations outside of the previously delineated polygon.

We generated sample points within the boundaries of delineated brood-use polygons using the “create random points” tool in ArcToolbox. Stratified sampling was not used for two reasons. First, information from a simple Landsat-based cover classification was available only from ME at the time and produced three cover classes within brood-use areas, one of which largely dominated polygons. Second, site visits indicated the two cover classes identified did a poor job of distinguishing alpine vegetation communities. More random points were generated than actually used per brood-use area. This was because several random points within each brood-use area were invariably in areas where sampling could not occur (e.g., streams, snowfields, boulder fields, trails). These points could not be identified prior to site visits based on available GIS data. Initially, a total of 10–30 random points were generated per brood-use area, depending on the area extent of the polygon. Points were visited in order of their identifying number and marked using two wood stakes placed at opposite corners of a 1-m^2^ sampling frame oriented so that sides were perpendicular to cardinal directions. In cases where sample points were located in areas void of vegetation (e.g., over a boulder or goat scrape), the frame was moved to the nearest vegetated area within 3 m of the point location. If the nearest point was > 3 m distance it was discarded and the next sampling point was visited. We justified moving sample points in these instances because our objective was to track changes in plant phenology and not to estimate different categories of ground cover or direct loss of vegetation.

A total of 36 sample points at ME and 39 at TR was established within brood-use areas. Sample points varied by year at MS from 27 in 2013, to 12 in 2014, and 17 in 2015. Variation in sample points at MS was due to two factors. First, in 2013 the initial defined brood-use area did not contain the total extent of locations used by broods. As a result, the defined brood-use area was revised for 2014 and 2015 sampling. Second, the number of points used at MS was too many for crews to reliably survey during site visits (primarily due the travel time required to access the site and the occurrence of afternoon monsoon storms). Thus, the number of monitored points was reduced in 2014 and 2015 to a manageable number.

## SM 2: Estimating plant productivity and phenology

Plots were visited at weekly intervals to collect information on changes in plant productivity and phenology. Plant productivity was recorded using a point-and-shoot digital camera (Canon ELPH 110) modified to capture light in both visible and near infrared (NIR) spectrums (camera modifications by MaxMax, Ltd. Carlstadt, NJ). A 1-m^2^ sampling frame was placed over the stakes during each sampling period to ensure consistency in the area surveyed. Each photo was taken from roughly 1.5 m directly above the sampling frame in a plane horizontal with the ground. Photographs were taken from the same orientation between 10:00 and 14:00 to standardize lighting conditions as much as possible; most plot photographs were taken within a one-hour window across all weeks. Prior to taking photographs, a 1-m^2^ sampling frame of 1.27-cm diameter PVC pipe was placed over the staked corners of the plot. Photographs were taken from the north side of the sample quadrant to reduce or eliminate shadows from the observer. White balance was set to “cloudy” for all photographs based on recommendations for outdoor shooting provided by the manufacturer (Maxmax, Ltd.). Photographs were taken by holding the camera perpendicular to the ground and centered over the plot, and “bracketing” the quadrant sampling frame in the viewfinder to fill as much of the photograph with the sample plot as possible (as opposed to the area outside the sample plot which was of no interest).

The modified cameras captured NIR and visible electromagnetic bands in separate channels, with NIR captured in the red channel, and visible light captured in blue and green channels. Parsing data into separate channels was done to estimate plant productivity using standard indices. The traditional normalized difference vegetation index (NDVI) is used to estimate the amount of live vegetation in an image and is calculated using the formula (Carlson and Ripley 1997):

$$NDVI=\frac{\left( NIR-Red \right)}{\left( NIR+Red \right)}$$

This formula was modified when using data collected in digital cameras because the traditional formula uses the blue spectrum for NIR. In contrast, since the digital cameras we used captured NIR in the red channel, in which case the formula can be modified as (MaxMax Ltd., personal communication):

$$NDVI=\frac{\left( NIR-Blue \right)}{\left( NIR+Blue \right)}$$

However, the company responsible for the modifications of our digital cameras recommended using both reflective channels (i.e., red and green) to enhance (thus ENDVI) discernibility of live and dead plant tissue, which leads to the formula we used for processing the photographs:

$$ENDVI=\frac{\left( \left( NIR+Green \right)-(2*Blue) \right)}{\left( \left( NIR+Green \right)+(2*Blue) \right)}$$

This equation differs from the previous by adding green to the reflective channel. The absorption channel (blue) was multiplied by two to balance the additional data from the green channel. Boegh et al. (2002) reported that ENDVI is more correlated with green leaf area index, and ENDVI is one of two products available from MODIS satellite imagery. Thus, ENDVI is a widely recognized index for plant biomass and phenology measurements.

Raw photographs collected required processing in a series of steps before they could be used to estimate temporal changes in plant productivity. Photographs were first cropped to the extent of the sampling frame. The slope of the ground varied by plot, and as a result, standard cropping could not be used because the resulting quadrant was not always square in dimensions when viewed from above. We used the perspective crop tool in Adobe Photoshop (Version CC 2015) to correct for this distortion; resulting images were reprocessed so that all sides of the photograph were the same length (Fig. S1). For the second step of processing, distortion-corrected photographs were processed using the program ImageJ (Schneider et al. 2012). Code used to process photographs was provided as a macro by the manufacturer (MaxMax, Ltd.). The macro processed raw red-green-blue (RBG) photographs collected at vegetation plots by extracting color values from each pixel, assigning them to an appropriate bin, using the ENDVI formula to calculate an ENDVI value for each pixel, and averaging all pixels within a photo (Fig. S2) to produce an average ENDVI value for a given photograph. The macros included several settings for photographic processing, including a setting specifying highest values for areas in the photograph that were plants, highest values for areas in the photographs that were not plants, and a correction factor (baseband correction), which shifted the calculated ENDVI up or down. We specified values of 1 and -1 for the maximum plant and non-plant pixels, and left the baseband correction factor equal to 0. These settings remained constant for all processed photographs because adjustments between photographs would lead to non-comparable average ENDVI values. Throughout the manuscript and figures we refer to ENDVI generically as “NDVI.”

We recorded the number of species in bloom during each weekly vegetation plot visit. A species was considered to be in bloom if a flower head was present and visible. The presence or absence of a flower in bloom was entered as a 1 (present) or 0 (absent) for every species recorded at each study site. The total number of species recorded across all sites and plots was large and included 78 plant species. We pooled some species belonging to the same genus together to boost samples, including three species of *Trifolium* (*T. dasyphyllum, T. nanum, and T. parryi*), and two species of *Polygonum* (*P. bistortoides and P. viviparum*). We investigated raw data to determine if these species were similar in their phenology (i.e., in bloom at roughly the same time) prior to pooling.

## SM 3: Arthropod sampling and estimation of temporal changes in abundance

Sampling of invertebrates was limited to ME (2013–2015) and MS (2013–2014). No samples were collected at TR due to restrictions under the Wilderness Act and lack of authorization from the U.S. National Park Service under the approved research permit for our project. Temporal changes in invertebrate abundance were measured by randomly selecting photo plots in brood-use areas and establishing a 20-m insect transect at each selected plot. Invertebrate sampling began as soon as the transect areas were free of snow which generally occurred between the 1^st^ and 3^rd^ week of June. Either 3 or 4 sticky aphid papers (10.2 x 17.8 cm; Seabright Laboratories, Emeryville, CA) were pinned to the ground with 5.1 cm roofing nails along transects. The number of aphid papers per transect varied because some transect locations were heavily impacted by alpine rodents, and aphid papers were often heavily chewed or completely removed. We attempted to adjust for these anticipated losses by adding an additional paper along problem transects while still accounting for areas of trap paper sample per unit of time. A total of 18 insect transects were monitored at weekly intervals at ME during each year of the study (six per brood site). Sampling was limited to four transects at MS due to time constraints by field crews; only one brood-use area was identified and sampled at MS. Location information for monitored transects is provided in Table S1. Aphid papers were removed and replaced every seven days along transects. Collected traps were wrapped in clear kitchen plastic wrap to keep invertebrates intact and to contain the adhesive glue from the traps. Collected aphid papers were stored in brown paper bags on a dry shelf until they could be processed. Aphid papers were processed by counting the number of individual invertebrates identified using a dissecting microscope and tally counter. We identified individuals to the lowest taxonomic Order; this was either family or genus in the majority of samples. A complete list of all taxa identified in transect aphid traps, including known taxa consumed by ptarmigan chicks, is presented in Table S2.

Aphid sticky papers were most effective in capturing flying insects, and inference on some taxonomic groups, such as ants, could not be made. Only seven taxonomic groups had samples sufficiently large to model temporal abundance (Table S2). Estimates of temporal invertebrate abundance were only available for a subset of sites and years in our study. Thus, they could not be incorporated as covariates for the nest and brood survival analysis in the full dataset due to missing data for TR (all years) and MS (2015).

Captured-insect densities for each taxonomic group were calculated for each transect and weekly interval by counting the number of individuals in each taxonomic group per paper *p* and summing over all papers within each transect *t* for a given week *w*. The total paper area (*A*) was calculated for each transect because the number of papers and proportion of paper intact could vary each week (primarily due to loss to rodents). The total count (*C*) was divided by the total paper area to get the captured-insect density (*D*) for each transect:

$$D_{t,w}=\frac{\sum_{p=1}^{M} C_{p,t,w}}{\sum_{p=1}^{M} A_{p,t,w}}$$

where *M* represents the number of papers collected at a given transect. Papers were generally collected every 7 days from the day they were first set out. However, weather or field logistics at times required that transects be visited either a day or two early or late, so the interval lengths could vary. Papers left in the field for longer or shorter periods have differing exposure periods over which insects can be captured. The average captured-insect density for each transect was divided by the number of days in the interval between the date set out and the date retrieved to account for variation in exposure. This was done to calculate average daily captured-insect density. The exposure interval (*I*), which was the number of days from the date set out to the date retrieved, was calculated for each transect to obtain the average daily transect density (*DTD*) for each weekly sample by dividing *D* by *I*:

$${DTD}_{t,w}=\frac{D_{t,w}}{I_{t,w}}$$

Thus, for every week of the season a *DTD* was calculated for each transect. Taxa-specific values of *DTD* may have been influenced by the person counting the aphid papers. In 2013 and 2014 this was done by experts in the College of Agricultural Sciences at Colorado State University. In 2015, G. T. Wann processed papers independently. The taxa identified in 2015 were the same as those found in 2013 and 2014, but counts for flies (species of Muscidae) and leafhoppers (species of Cicadellidae) were roughly two times higher than counts from 2013 and 2014. This may have reflected a true difference in abundance in 2015, but it may also have been due to differences in counting technique (e.g., counting heads of flies versus wings). This indicates total abundance may not be comparable among years, but counts were done consistently by observers within years. Thus, temporal changes between years were still comparable. *DTD* was standardized for each year *y* and site *s* by dividing by the maximum *DTD* value for a given taxon. Thus, the standardized (*S*) value of *DTD* used in analysis was:

$${SI}_{t,w}=\frac{{DTD}_{t,w}}{MAX({DTD}_{s,y})}$$

The values of *SI* were used to estimate temporal changes in abundance by modeling them as a response variable in generalized additive models (GAMs). The mid-point of the sampling interval (recorded as Julian day) was used as the explanatory variable in GAM models over which changes in *S* was predicted. Using the mid-point assumed that aphid papers captured insects at a fixed rate for each day in the sample. This was likely not true since aphid papers may capture insects at higher rates soon after they are set out. However, this was unlikely to be problematic since capture rates probably declined at similar rates among all transects (i.e., transects were still comparable). It is important to note that *SI* is a measure of captured invertebrate densities along individual transects. However, because transects were sampled randomly within brood-use areas, the average of each *S* within a given brood-use area and sampling period can be considered representative of the densities of captured taxa over the entire brood-use area.

## SM 4: Cross-correlations to examine relationships between two time series

We examined the relationships between temporal abundance of different invertebrate and plant taxa and how they related to average NDVI at ME (Fig. S3) and MS (Fig. S4). Cross-correlation functions (CCF) were used to examine the relationships between timing of plant and insect abundance. Cross-correlation functions are useful for identifying the correlation between two time series at different time lags. Considering time lags in comparisons between two time series is important because one series may be dependent on the other, in which case the dependent time series may have a delayed response. It is also possible that both time series being compared are responding to the same environmental stimulus, but the rate or timing of response could differ. CCFs provide a way to identify at what point in time two series are most correlated.

Cross-correlations were calculated using the stats package and ccf function in R. Predictions from the generalized additive models were the vectors for each time series. Every cross-correlation calculation included NDVI as one vector, which was our proxy for plant productivity, and a second vector, which was either plant-specific probabilities of bloom or taxa-specific predictions of insect abundance. Predictions from generalized additive models were smoothed, which resulted in cross-correlation relationships that were also smooth in appearance. Generally, the relationship between NDVI was consistent across both years and sites for insect taxa of known food quality for ptarmigan (ME, Fig. S5 and MS, Fig. S7). Relationships were highly consistent for insect taxa of unknown forage quality (ME, Fig. S6 and MS, Fig. S8). The consistencies in the relationships between NDVI and bloom of forage species were extremely similar across all sites and years (ME, Fig. S9; TR, Fig. S10; and MS, Fig. S11). Two species that bloomed early, late, or at the same time as plants reached peak biomass did so at similar time lags across sites and years; these were *Polygonum* spp. and *Geum rossii*, two of the dominant forbs at our study sites. Species of *Trifolium* were the earliest to bloom, but both *Trifolium* and *Artemisia* species were inconsistent in terms of their bloom relative to NDVI. ME had the most consistent relationships between plant bloom and NDVI among years, followed by TR and MS.

Invertebrate abundance was variable among years for species of known food quality, although the relationships were remarkably consistent for all years at ME for Muscidae (flies) and Acrididae (grasshoppers). The variability in MS data was higher than at ME (Table S4.2). This was likely related to differences in annual sample sizes at both sites, which were about four times greater at ME. However, there were general observable patterns at both sites, and flies tended to peak close to the same time as NDVI. The remarkable correlations were for invertebrates of unknown forage quality, which consisted of species in the families Paplionidae (composed entirely of *Parnassius smintheus* samples), Nymphalidae (composed entirely of *Bolaria* genus samples), and Pieridae (composed entirely of *Colias* *meadii* samples). The peaks in Nymphalidae at ME closely coincided with peak NDVI, and the peaks for Paplionidae and Pieridae occurred after peak NDIV. The time lags at peak correlation varied little among years at ME, suggesting NDVI was a strong predictor of peak abundance for these species. Overall, for our site with the largest invertebrate sample sizes (ME), we were able to model relationships of invertebrate abundance that showed clear patterns in daily changes across the breeding season. Correlations between abundance of invertebrates and NDVI indicate that changes in above ground plant productivity (i.e., above ground biomass) is likely a useful metric to predict changes in abundance for the invertebrate we sampled.

## SM 5: Model assumptions and development

One assumption of the young-survival model is that young cannot be added to broods (i.e., no chick adoption) because adoptions may positively or negatively bias survival estimates (Lukacs et al. 2004). This assumption was likely violated at our study sites because ptarmigan are known to adopt young from other broods at rates ranging from 4-14% (Wong et al. 2009). Mixed broods (i.e., 2 hens together with chicks) were sometimes observed at our sites, but identifying the correct hen to assign chicks to was generally straightforward due to size differences between chicks of different ages, and because most broods were captured and banded once they reached the age of 4 to 5 weeks. On 1% of occasions (14 out of 1211 encounters), single-hen broods less than 5 weeks old were observed to gain chicks of a different size between counts. In such cases, we excluded the additional chick(s) in the count for the encounter occasion they were observed on. Doing so had negligible effects on our candidate model set (i.e., model rankings remained the same and model support was essentially unchanged compared to leaving those chicks in the encounter history).

# Literature cited

Boegh, E., H. Soegaard, N. Broge, C. B. Hasager, N. O. Jensen, K. Schelde, and A. Thomsen. 2002. Airborne multispectral data for quantifying leaf area index, nitrogen concentration, and photosynthetic efficiency in agriculture. Remote Sensing of Environment 81:179–193.

Lukacs, P. M., V. J. Dreitz, F. L. Knopf, and K. P. Burnham. 2004. Estimating survival probabilities of unmarked dependent young when detection is imperfect. Condor 106:926–931.

Schneider, C. A., W. S. Rasband, and K. W. Eliceiri. 2012. NIH Image to ImageJ: 25 years of image analysis. Nature Methods 9:671–675.

Wong, M. M. L., B. C. Fedy, S. Wilson, and K. M. Martin. 2009. Adoption in rock and white-tailed ptarmigan. Wilson Journal of Ornithology 121:638–641.

# Supplementary Tables

**Table S1**. Locations of insect transects at Mt. Evans (ME; 2013–2014) in Clear Creek County, Colorado, USA and Mesa Seco (MS; 2013 and 2014) in Hinsdale County, Colorado, USA. Subsites designate brood-use areas within study sites (there was only one delineated brood-use area at Mesa Seco during both years). UTM easting and northing were recorded in the NAD 83 datum (zone 13N).

| Site | Sub site | Years | Transect ID | Easting | Northing | Elevation (m) |
| --- | --- | --- | --- | --- | --- | --- |
| ME | Mt. Evans - NE | 2013-2015 | T05 | 445387 | 4382578 | 4007 |
| ME | Mt. Evans - NE | 2013-2015 | T06 | 445510 | 4382162 | 4107 |
| ME | Mt. Evans - NE | 2013-2015 | T08 | 445755 | 4382153 | 3993 |
| ME | Mt. Evans - NE | 2013-2015 | T23 | 445662 | 4381877 | 4090 |
| ME | Mt. Evans - NE | 2013-2015 | T24 | 445311 | 4382537 | 4032 |
| ME | Mt. Evans - NE | 2013-2015 | T25 | 445858 | 4381839 | 4047 |
| ME | Mt. Warren | 2013-2015 | T26 | 446376 | 4383739 | 3886 |
| ME | Mt. Warren | 2013-2015 | T32 | 446394 | 4383928 | 3864 |
| ME | Mt. Warren | 2013-2015 | T36 | 446075 | 4384251 | 3952 |
| ME | Mt. Warren | 2013-2015 | T41 | 445909 | 4384263 | 3998 |
| ME | Mt. Warren | 2013-2015 | T42 | 446498 | 4383620 | 3824 |
| ME | Mt. Warren | 2013-2015 | T43 | 446377 | 4383615 | 3843 |
| ME | Mt. Rogers | 2013-2015 | T51 | 447526 | 4385426 | 3932 |
| ME | Mt. Rogers | 2013-2015 | T60 | 447360 | 4385450 | 3985 |
| ME | Mt. Rogers | 2013-2015 | T61 | 447481 | 4385701 | 3957 |
| ME | Mt. Rogers | 2013-2015 | T63 | 447417 | 4385347 | 3932 |
| ME | Mt. Rogers | 2013-2015 | T70 | 447402 | 4385415 | 3956 |
| ME | Mt. Rogers | 2013-2015 | T72 | 447483 | 4385167 | 3868 |
| MS | Mesa Seco | 2013 | T1 | 303261 | 4212165 | 3854 |
| MS | Mesa Seco | 2013 | T2 | 303217 | 4211925 | 3889 |
| MS | Mesa Seco | 2013 | T3 | 303244 | 4211598 | 3877 |
| MS | Mesa Seco | 2013 | T4 | 303245 | 4211297 | 3848 |
| MS | Mesa Seco | 2014 | T1 | 303022 | 4212121 | 3894 |
| MS | Mesa Seco | 2014 | T2 | 303546 | 4212466 | 3793 |
| MS | Mesa Seco | 2014 | T3 | 303399 | 4211570 | 3854 |
| MS | Mesa Seco | 2014 | T4 | 303729 | 4210773 | 3814 |

**Table S2**. Invertebrate taxon identified from aphid paper traps at Mt. Evans in Clear Creek County, Colorado, USA (2013–2015), and Mesa Seco in Hinsdale County, Colorado, USA (2013–2014). Rows marked with an asterisk (*) represent a taxa of known dietary importance for white-tailed ptarmigan (*Lagopus leucura*) chicks, either through crop content inspection, or visually observing chicks consume invertebrates. Rows marked with “^” represent invertebrates from a taxonomic class other than Insecta. NA = could not be identified to taxonomic level.

| Order | Family | Genus | Species |
| --- | --- | --- | --- |
| Araneae^*,^^ | NA | NA | NA |
| Coleoptera^*^ | Carabidae | NA | NA |
| Coleoptera | Carabidae | Pterostichus | NA |
| Diptera | Bombyliidae | NA | NA |
| Diptera^*^ | Muscidae | NA | NA |
| Diptera | Phoridae | NA | NA |
| Diptera | Sciaridae | NA | NA |
| Diptera | Syrphidae | NA | NA |
| Diptera^*^ | Tipulidae | NA | NA |
| Hemiptera | Cicadellidae | NA | NA |
| Hymenoptera | Apidae | NA | NA |
| Hymenoptera | Cynipidae | NA | NA |
| Hymenoptera | Eulophidae | NA | NA |
| Hymenoptera^*^ | Formicidae | NA | NA |
| Hymenoptera | Ichneumonidae | NA | NA |
| Hymenoptera | Tenthredinidae | NA | NA |
| Lepidoptera | Geometridae | NA | NA |
| Lepidoptera | Hesperiidae | NA | NA |
| Lepidoptera | Noctuidae | NA | NA |
| Lepidoptera | Nymphalidae | *Aglais* | *milberti* |
| Lepidoptera | Nymphalidae | *Boloria* | NA |
| Lepidoptera | Nymphalidae | NA | NA |
| Lepidoptera | Papilionidae | *Parnassius* | *smintheus* |
| Lepidoptera | Pieridae | *Colias* | *meadii* |
| Lepidoptera | Saturniidae | *Hemileuca* | NA |
| Lepidoptera | Sphingidae | NA | NA |
| Orthoptera^*^ | Acrididae | *Aeropedellus* | *clavatus* |
| Orthoptera | Tettigoniidae | *Anabrus* | *simplex* |

**Table S3**. Time lags and cross correlations between NDVI and invertebrates for three Colorado study sites. Locations were Mt. Evans (ME) in Clear Creek County, Trail Ridge (TR) in Larimer County, and Mesa Seco (MS) in Hinsdale County. Time lags represent the lag (in days) when positive correlations were the highest. Negative time lags indicate when invertebrate abundance preceded peak NDVI, while positive lags indicate when invertebrate abundance occurred after peak NDVI. Average values for each site are provided at the bottom row of each species comparison, and average values by site are provided in the two leftmost columns.

| Comparison | Year | Lag ME | Lag MS | Cor. ME | Cor. MS | Ave. Lag | Ave. Cor. |
| --- | --- | --- | --- | --- | --- | --- | --- |
| Muscidae | 2013 | 0 | -19 | 0.815 | 0.309 | **-9.5** | **0.562** |
| Muscidae | 2014 | -10 | 0 | 0.463 | 0.88 | **-5** | **0.672** |
| Muscidae | 2015 | -29 | NA | 0.284 | NA | **-29** | **0.284** |
| **Ave. Muscidae** |  | **-13.0** | **-9.5** | **0.521** | **0.595** |  |  |
| Acrididae | 2013 | 8 | 0 | 0.769 | 0.867 | **4** | **0.818** |
| Acrididae | 2014 | 29 | 0 | 0.557 | 0.917 | **14.5** | **0.737** |
| Acrididae | 2015 | 0 | NA | 0.851 | NA | **0** | **0.851** |
| **Ave. Acrididae** |  | **12.3** | **0** | **0.726** | **0.892** |  |  |
| Cicadellidae | 2013 | -38 | 0 | 0.452 | 0.984 | **-19** | **0.718** |
| Cicadellidae | 2014 | -33 | 0 | 0.426 | 0.363 | **-16.5** | **0.395** |
| Cicadellidae | 2015 | -40 | NA | 0.313 | NA | **-40** | **0.313** |
| **Ave. Cicadellidae** |  | **-37.0** | **0** | **0.397** | **0.674** |  |  |
| Papilionidae | 2013 | 27 | 0 | 0.677 | 0.863 | **13.5** | **0.770** |
| Papilionidae | 2014 | 31 | 0 | 0.61 | 0.917 | **15.5** | **0.764** |
| Papilionidae | 2015 | 28 | NA | 0.531 | NA | **28** | **0.531** |
| **Ave. Papilionidae** |  | **28.7** | **0** | **0.606** | **0.89** |  |  |
| Nymphalidae | 2013 | 0 | -34 | 0.89 | 0.404 | **-17** | **0.647** |
| Nymphalidae | 2014 | 2 | 0 | 0.891 | 0.522 | **1** | **0.707** |
| Nymphalidae | 2015 | 0 | NA | 0.755 | NA | **0** | **0.755** |
| **Ave. Nymphalidae** |  | **0.7** | **-17.0** | **0.845** | **0.463** |  |  |
| Pieridae | 2013 | 26 | 0 | 0.777 | 0.35 | **13** | **0.564** |
| Pieridae | 2014 | 29 | 0 | 0.639 | 0.695 | **14.5** | **0.667** |
| Pieridae | 2015 | 27 | NA | 0.508 | NA | **27** | **0.508** |
| **Ave. Pieridae** |  | **27.3** | **0.0** | **0.641** | **0.523** |  |  |

**Table S4**. Time lags and cross correlations between NDVI and plant bloom for three Colorado study sites. Locations were Mt. Evans (ME) in Clear Creek County, Trail Ridge (TR) in Larimer County, and Mesa Seco (MS) in Hinsdale County. Time lags represent the lag (in days) when positive correlations were the highest. Negative time lags indicate when onset of bloom preceded peak NDVI, while positive lags indicate when bloom occurred after peak NDVI. Average values for each site are provided at the bottom row of each species comparison, and average values by site are provided in the two right columns.

| Comparison | Year | Lag ME | Lag TR | Lag MS | Cor. ME | Cor. TR | Cor. MS | Ave. Lag | Ave. Cor. |
| --- | --- | --- | --- | --- | --- | --- | --- | --- | --- |
| *Trifolium* spp. | 2013 | 0 | -5 | -34 | 0.558 | 0.453 | 0.254 | **-13** | **0.422** |
| *Trifolium* spp. | 2014 | -8 | -40 | -40 | 0.578 | 0.292 | 0.448 | **-29** | **0.439** |
| *Trifolium* spp. | 2015 | -5 | -34 | -33 | 0.453 | 0.254 | 0.295 | **-24** | **0.334** |
| **Ave. *Trifolium* spp.** |  | **-4.3** | **-26.3** | **-35.7** | **0.530** | **0.333** | **0.332** |  |  |
| *Geum rossii* | 2013 | -6 | -26 | -5 | 0.555 | 0.334 | 0.289 | **-12** | **0.393** |
| *Geum rossii* | 2014 | 0 | -37 | -21 | 0.674 | 0.282 | 0.328 | **-19** | **0.428** |
| *Geum rossii* | 2015 | 0 | 0 | -5 | 0.667 | 0.717 | 0.289 | **-2** | **0.558** |
| **Ave. *Geum rossii*** |  | **-2.0** | **-21.0** | **-10.3** | **0.632** | **0.444** | **0.302** |  |  |
| *Polygonum* spp. | 2013 | 0 | -2 | -14 | 0.967 | 0.590 | 0.884 | **-5** | **0.814** |
| *Polygonum* spp. | 2014 | 3 | -3 | 0 | 0.837 | 0.596 | 0.619 | **0** | **0.684** |
| *Polygonum* spp. | 2015 | 0 | -7 | 0 | 0.956 | 0.416 | 0.550 | **-2** | **0.641** |
| **Ave. *Polygonum* spp.** |  | **1.0** | **-4.0** | **-4.7** | **0.920** | **0.534** | **0.684** |  |  |
| *Artemisia* spp. | 2013 | -2 | -17 | -40 | 0.765 | 0.315 | 0.465 | **-19.7** | **0.515** |
| *Artemisia* spp. | 2014 | 12 | -31 | -25 | 0.733 | 0.316 | 0.316 | **-14.7** | **0.455** |
| *Artemisia* spp. | 2015 | 0 | 0 | 0 | 0.626 | 0.939 | 0.229 | **0** | **0.598** |
| **Ave. *Artemisia* spp.** |  | 3.3 | -16.0 | -21.7 | 0.708 | 0.523 | 0.337 |  |  |

**Table S5**. Full model selection results for top brood survival models fit to data collected from white-tailed ptarmigan (*Lagopus leucura*) populations from 2013–2015 in Colorado, USA. The difference between AICc from the top model (Δ AICc), model support (AICc Weight), model likelihood, number of parameters (*K*), and model deviance are shown. Parameters in the model were apparent survival of chicks (ϕ) and recapture probability (*p*). Group effects and covariates included study site (site), year of study (year), chick age (CAGE), minimum and maximum temperatures (min and max, respectively), precipitation (precip), time difference mismatch (TDM), area mismatch calculated from average NDVI curve (SeasM), NDVI at hatch (PostM), and mismatch for species-specific plant curves, including *Artemisia* (ArteM), *Trifolium* (TrifM*), Geum* (GeumM), *Polygonum* (PolyM) and the cumulative are under each forb curve combined (ForbM). All model structures included the base structure that received highest support during the first stage of model selection {b0 + site + year + CAGE + site x year + site x CAGE}, represented as “best” in model formulas.

| Model | Δ AICc | AICc Weights | Likelihood | K | Deviance |
| --- | --- | --- | --- | --- | --- |
| ϕ(best + SeasM + CAGE x SeasM)*p*(site) | 0.0 | 0.93 | 1.00 | 17 | 1622.3 |
| ϕ(best + precip)*p*(site) | 8.2 | 0.02 | 0.02 | 16 | 1632.6 |
| ϕ(best +ForbM + CAGE x ForbM)*p*(site) | 8.8 | 0.01 | 0.01 | 17 | 1631.1 |
| ϕ(best + min)*p*(site) | 9.7 | 0.01 | 0.01 | 16 | 1634.1 |
| ϕ(best + precip + CAGE x precip)*p*(site) | 10.3 | 0.01 | 0.01 | 17 | 1632.5 |
| ϕ(best + SeasM)*p*(site) | 10.3 | 0.01 | 0.01 | 16 | 1634.7 |
| ϕ(best + TrifM)*p*(site) | 11.8 | 0.00 | 0.00 | 16 | 1636.2 |
| ϕ(best + min + CAGE x min)*p*(site) | 11.8 | 0.00 | 0.00 | 17 | 1634.1 |
| ϕ(best)*p*(site) | 12.2 | 0.00 | 0.00 | 15 | 1638.7 |
| ϕ(best + ArteM)*p*(site) | 12.6 | 0.00 | 0.00 | 16 | 1637.0 |
| ϕ(best + max)*p*(site) | 12.8 | 0.00 | 0.00 | 16 | 1637.1 |
| ϕ(best + GeumM)*p*(site) | 12.9 | 0.00 | 0.00 | 16 | 1637.3 |
| ϕ(best + TDM)*p*(site) | 13.3 | 0.00 | 0.00 | 16 | 1637.7 |
| ϕ(best + PostM)*p*(site) | 13.5 | 0.00 | 0.00 | 16 | 1637.9 |
| ϕ(best + PolyM)*p*(site) | 13.7 | 0.00 | 0.00 | 16 | 1638.1 |
| ϕ(best + ForbM)*p*(site) | 13.7 | 0.00 | 0.00 | 16 | 1638.1 |
| ϕ(best + TrifM + CAGE x TrifM)*p*(site) | 13.8 | 0.00 | 0.00 | 17 | 1636.1 |
| ϕ(best + TDM + CAGE x TDM)*p*(site) | 14.3 | 0.00 | 0.00 | 17 | 1636.6 |
| ϕ(best + ArteM + CAGE x ArteM)*p*(site) | 14.7 | 0.00 | 0.00 | 17 | 1637.0 |
| ϕ(best + max + CAGE x max)*p*(site) | 14.7 | 0.00 | 0.00 | 17 | 1637.0 |
| ϕ(best + GeumM + CAGE x GeumM)*p*(site) | 14.9 | 0.00 | 0.00 | 17 | 1637.2 |
| ϕ(best + PostM + CAGE x PostM)*p*(site) | 15.2 | 0.00 | 0.00 | 17 | 1637.5 |
| ϕ(best + PolyM + CAGE x PolyM)*p*(site) | 15.5 | 0.00 | 0.00 | 17 | 1637.8 |

**Table S6**. Predicted apparent daily survival rates (ϕ) for 3-day-old white-tailed ptarmigan (*Lagopus leucura*) chicks studied at three sites in Colorado, USA from 2013–2015. Study sites were at Mt. Evans (ME) in Clear Creek County, Mesa Seco (MS) in Hinsdale County, and Trail Ridge (TR) in Larimer County. Predictions are for survival rates when the area under the phenology curve between hatch and end of season was 1 (low mismatch) or 6 (high mismatch). Lower (LCL) and upper (UCL) 95% confidence intervals are provided for each point estimate, and the percent decline in survival from low to high mismatch predictions are provided on the bottom three rows.

|  |  | 2013 |  |  | 2014 |  |  | 2015 |  |
| --- | --- | --- | --- | --- | --- | --- | --- | --- | --- |
| Sites | ϕ | LCL | UCL | ϕ | LCL | UCL | ϕ | LCL | UCL |
| *Mismatch = 1* |  |  |  |  |  |  |  |  |  |
| ME | 0.986 | 0.954 | 0.996 | 0.984 | 0.951 | 0.995 | 0.982 | 0.952 | 0.993 |
| MS | 0.850 | 0.769 | 0.906 | 0.953 | 0.916 | 0.974 | 0.947 | 0.843 | 0.984 |
| TR | 0.993 | 0.981 | 0.997 | 0.991 | 0.975 | 0.997 | 0.984 | 0.960 | 0.994 |
| *Mismatch = 6* |  |  |  |  |  |  |  |  |  |
| ME | 0.913 | 0.869 | 0.943 | 0.898 | 0.859 | 0.928 | 0.887 | 0.809 | 0.936 |
| MS | 0.450 | 0.179 | 0.753 | 0.743 | 0.513 | 0.889 | 0.722 | 0.601 | 0.818 |
| TR | 0.956 | 0.905 | 0.980 | 0.940 | 0.878 | 0.972 | 0.899 | 0.825 | 0.944 |
| *Percent change* | |  |  |  |  |  |  |  |  |
| ME | -7.4 | - | - | -8.7 | - | - | -9.7 | - | - |
| MS | -47.1 | - | - | -22.0 | - | - | -23.8 | - | - |
| TR | -3.7 | - | - | -5.1 | - | - | -8.7 | - | - |

# Supplementary Figures


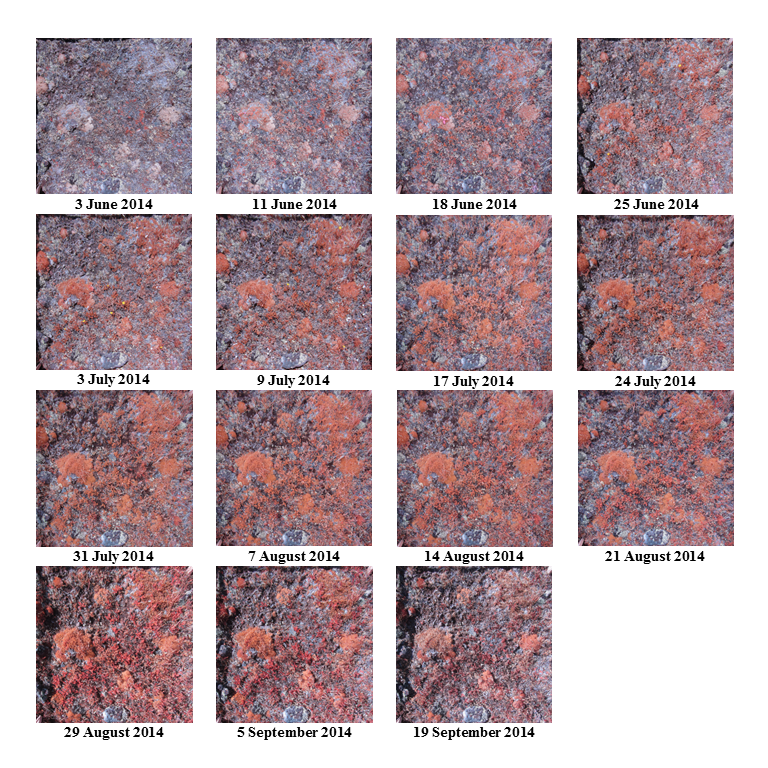


**Figure S1**. Series of pre-NDVI processed photographs taken at one sample point in 2014 in Trail Ridge (TR), Larimer County, Colorado. All photographs in the series are shown in raw format prior to NDVI processing. Photographs are cropped to the 1-m^2^ sample frame extent with a distortion correction.


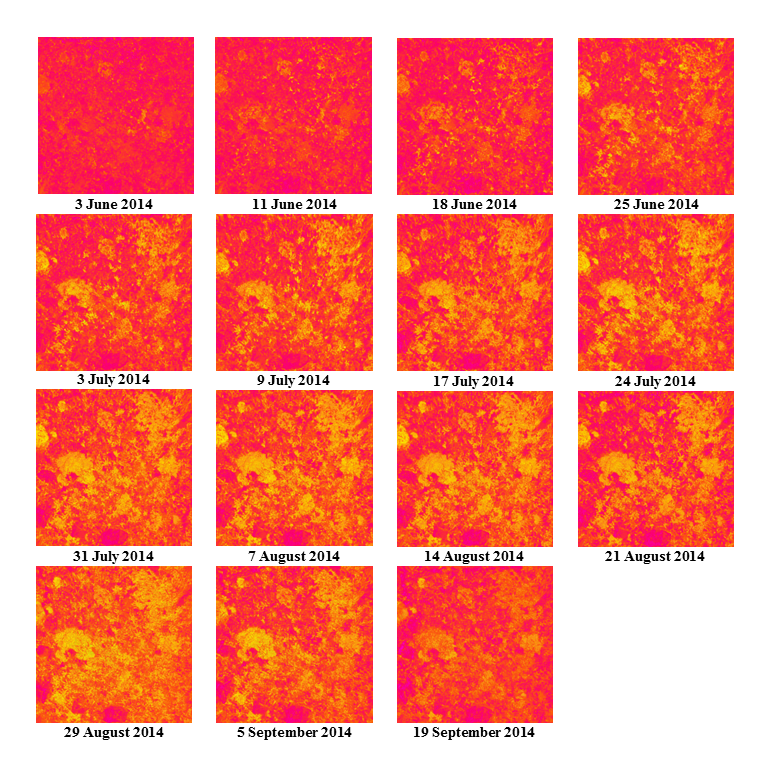


**Figure S2**. Series of post-NDVI processed photographs taken at one sample point in 2014 in Trail Ridge (TR), Larimer County, Colorado. Photographs in this series are the same as in Fig. S1. Regions of the photographs that are red or magenta represent dead or absent vegetation. In contrast, areas yellow or light green represent live vegetation. Each pixel has an associated NDVI value which is averaged with all other pixels to calculate an NDVI value for each photograph.


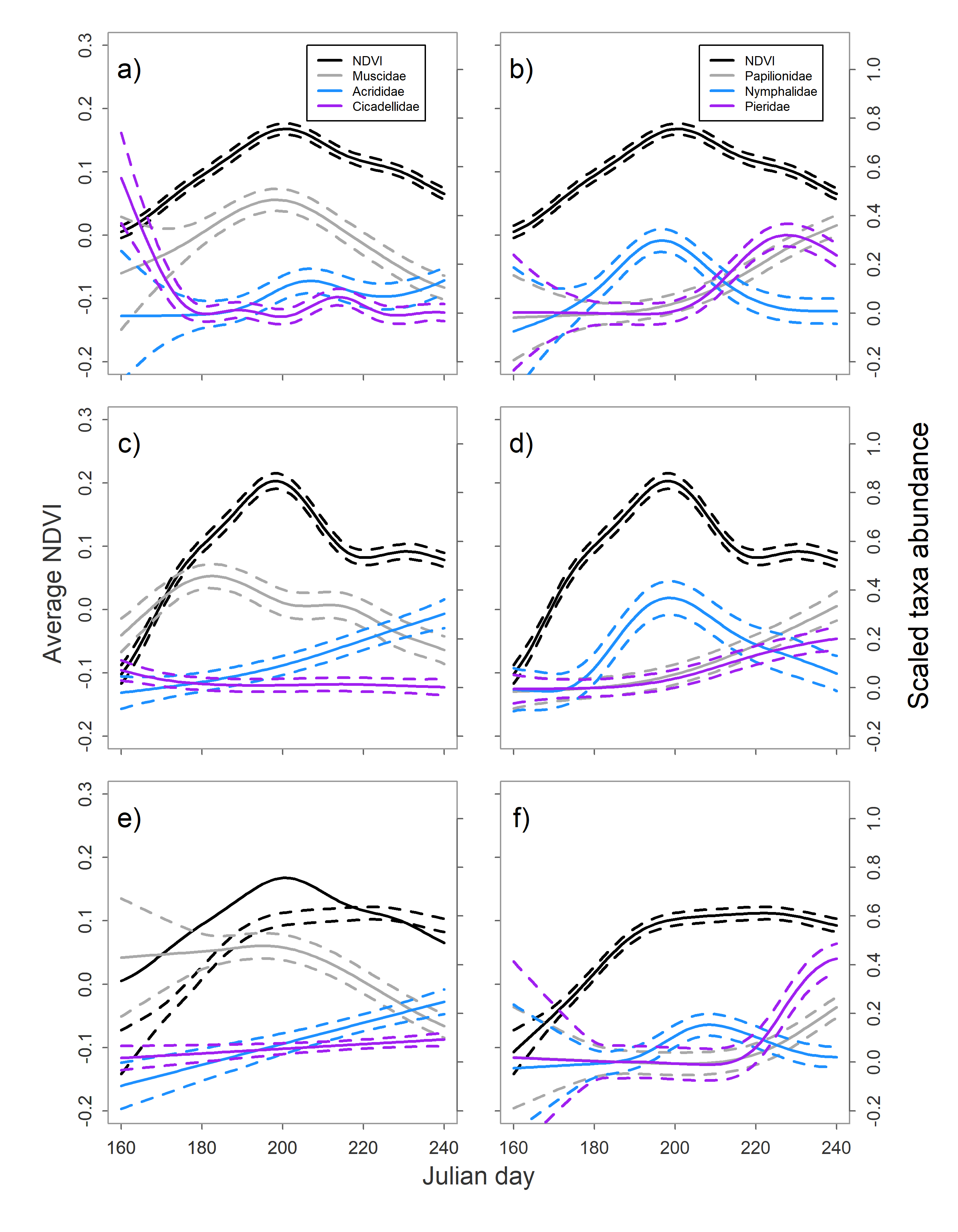


**Figure S3**. Temporal abundance of plant productivity (NDVI, left y-axis) and invertebrates (scaled taxa abundance, right y-axis) at Mt. Evans (ME) in Clear Creek County, Colorado, USA. Food invertebrates are in the left column for 2013–2015 (panels a, c, and e, respectively), and invertebrates of unknown food quality are in the right column for the same years (panels b, d, and f, respectively). Solid lines represent predictions from a generalized additive model and dashed lines are the associated 95% confidence intervals.

**
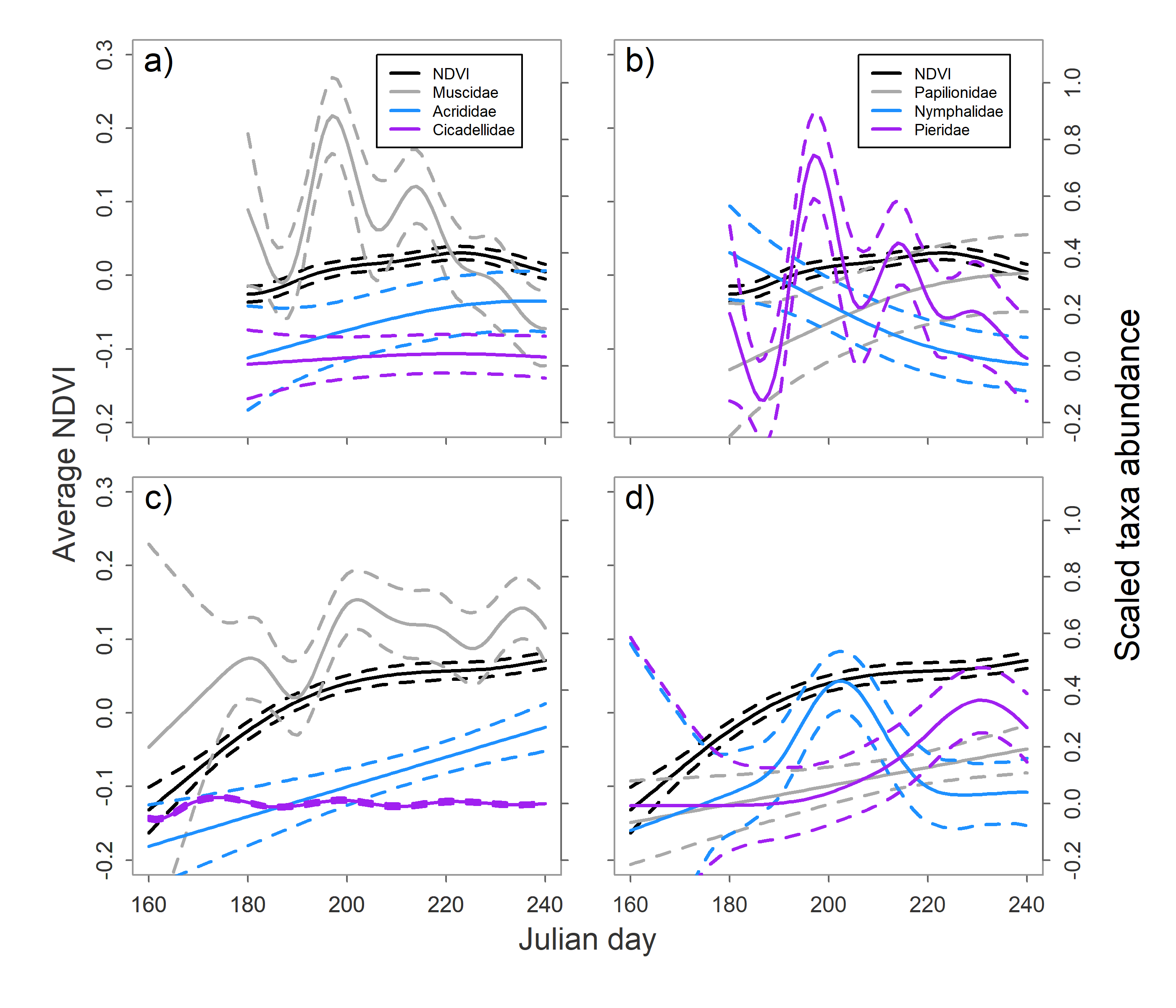
**

**Figure S4**. Temporal abundance of plant productivity (NDVI, left y-axis) and invertebrates (scaled taxa abundance, right y-axis) at Mesa Seco (MS) in Hinsdale County, Colorado, USA. Food invertebrates are in the left column for 2013–2014 (panels a and c, respectively), and invertebrates of unknown food quality are in the right column for the same years (panels b and d, respectively). Solid lines represent predictions from a generalized additive model and dashed lines are the associated 95% confidence intervals. Sampling was not completed at MS in 2015.


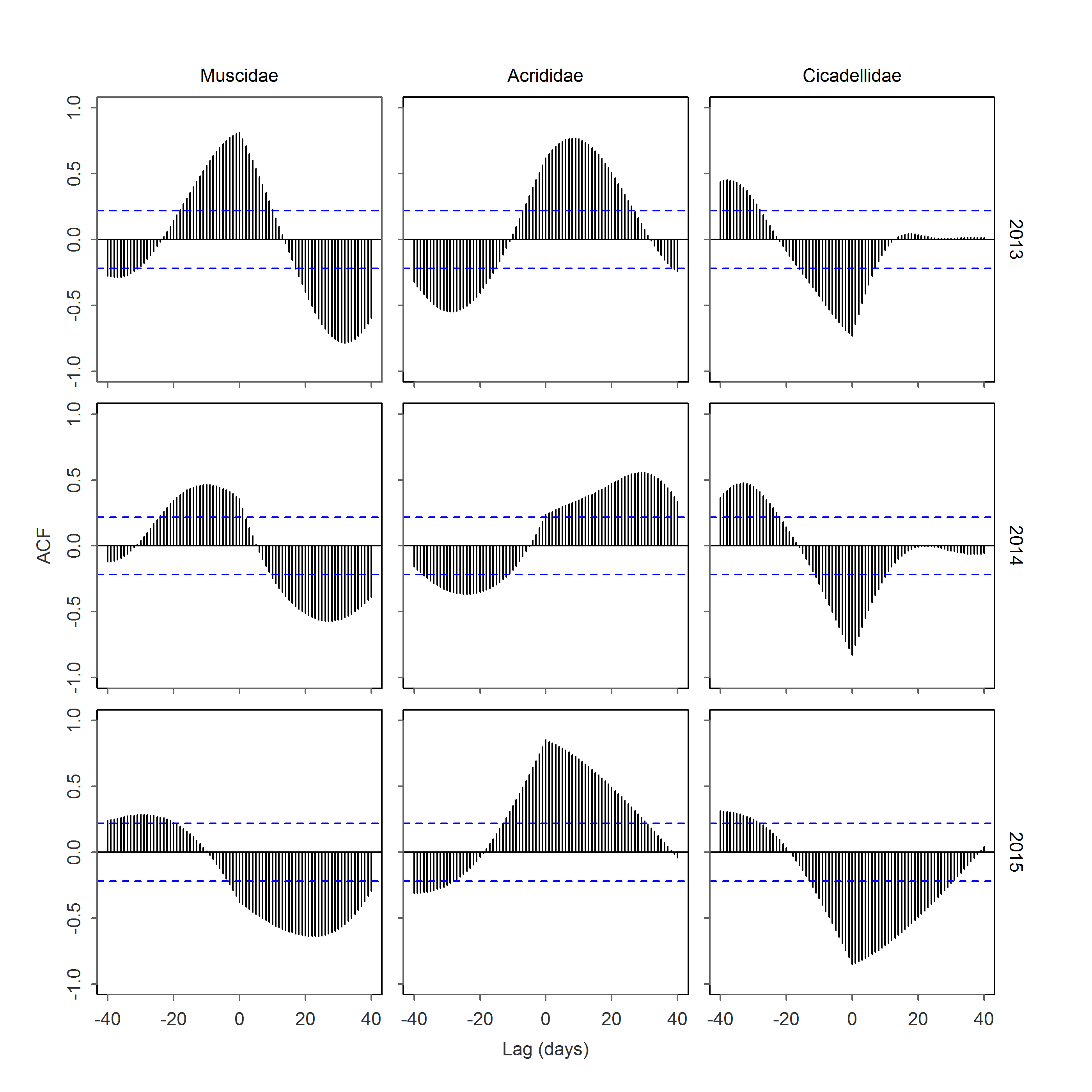


**Figure S5**. Cross-correlations for two temporally-overlapping time series (invertebrate food taxa and NDVI) at Mt. Evans (ME) in Clear Creek County, Colorado, USA. The y-axis represents the autocorrelation function at different lagged values in days (x-axis). The autocorrelation function is calculated between plant productivity values (NDVI) and invertebrate taxa for each year of the study (columns represent specific taxa and rows represent year). The blue dashed lines represent 95% confidence intervals beyond which autocorrelations are statistically different from zero. Negative lag values with positive correlations indicate correlations when invertebrates lead NDVI, and positive lag values indicate correlations when NDVI leads invertebrates.


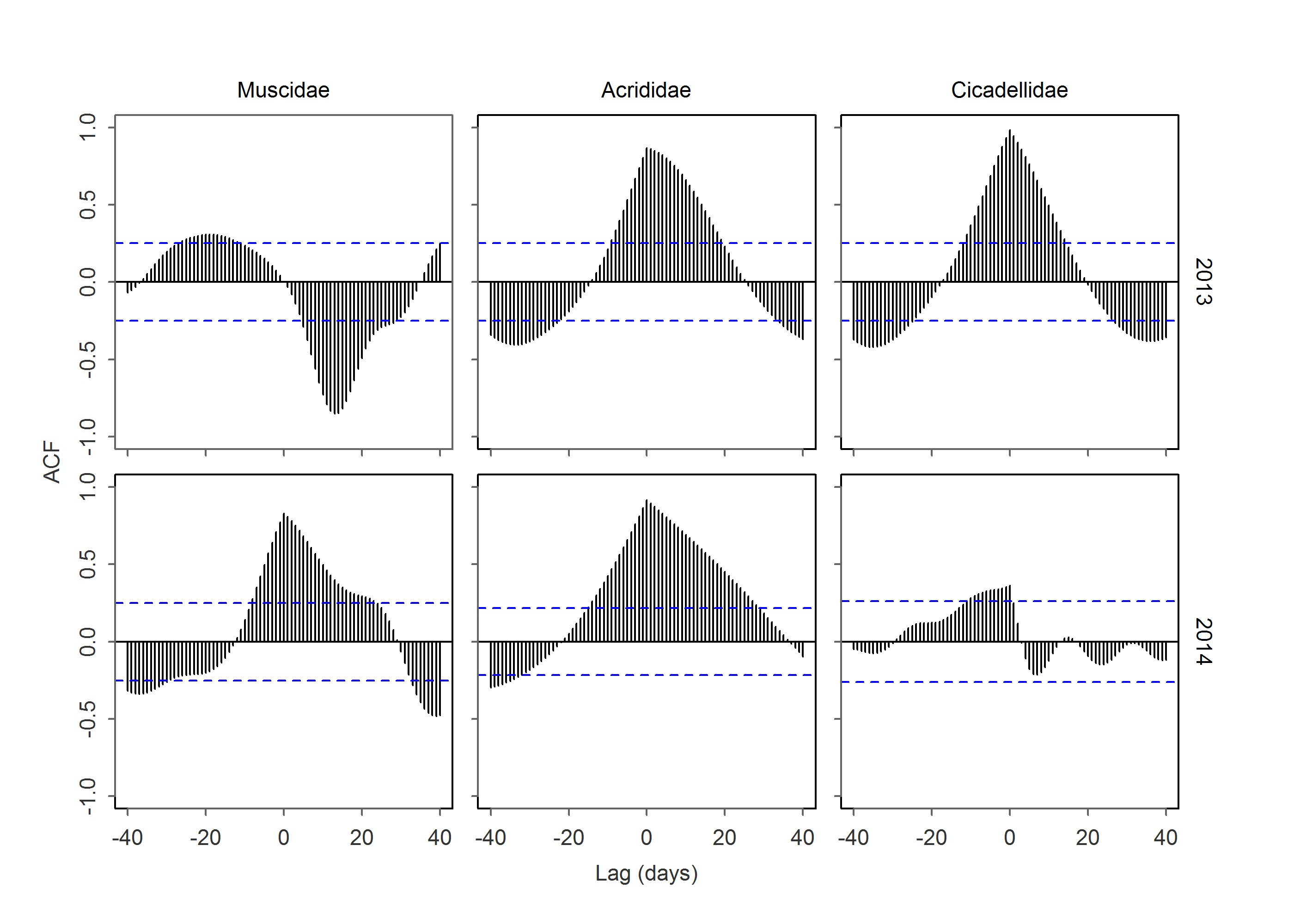


**Figure S6**. Cross-correlations for two temporally-overlapping time series (invertebrate food taxa and NDVI) at Mesa Seco (MS) in Hinsdale County, Colorado, USA. The y-axis represents the autocorrelation function at different lagged values in days (x-axis). The autocorrelation function is calculated between plant productivity values (NDVI) and invertebrate taxa for each year of the study (columns represent specific taxa and rows represent year). The blue dashed lines represent 95% confidence intervals beyond which autocorrelations are statistically different from zero. Negative lag values with positive correlations indicate correlations when invertebrates led NDVI, and positive lag values indicate correlations when NDVI led invertebrates.

**
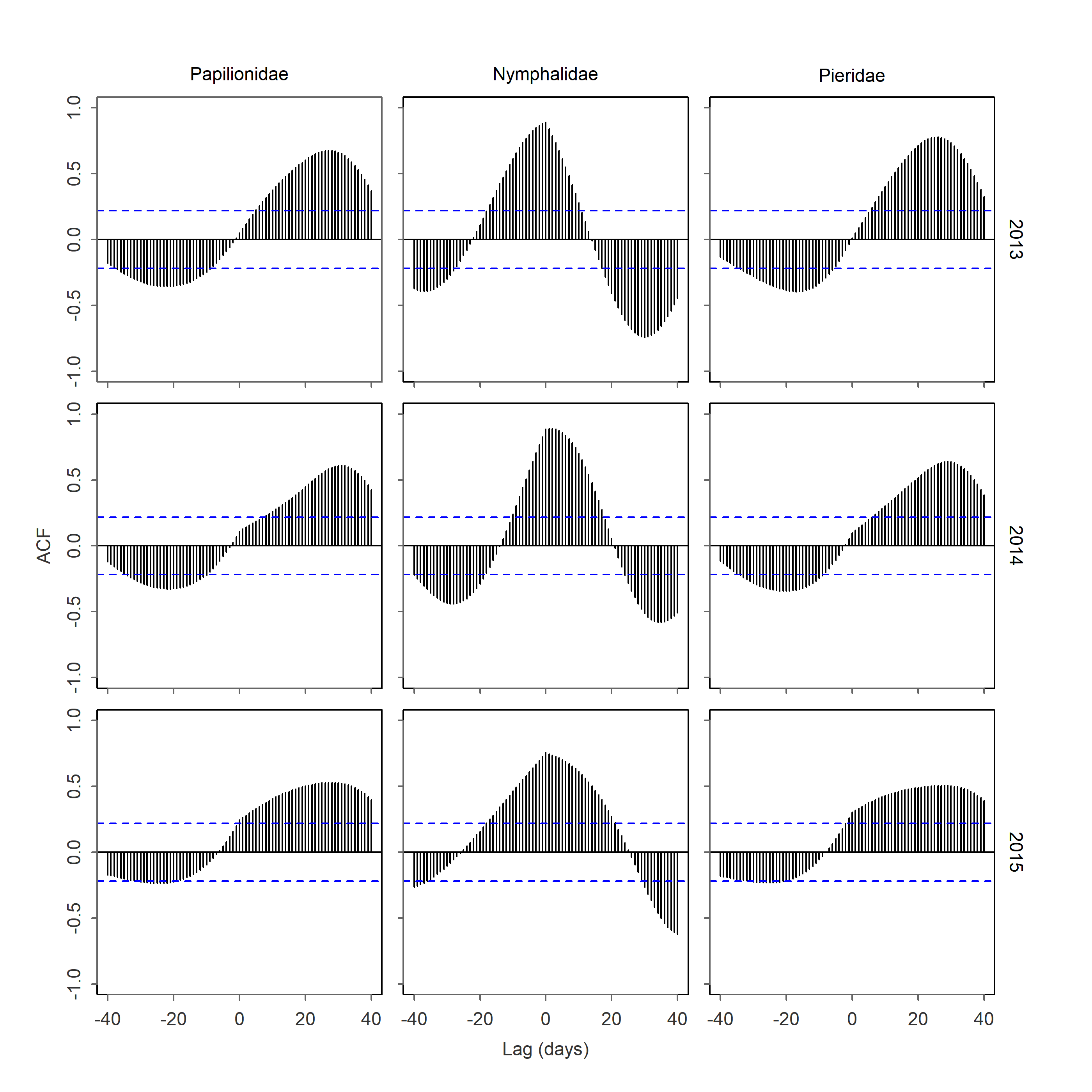
**

**Figure S7**. Cross-correlations for two temporally-overlapping time series (invertebrate food taxa and NDVI) at Mt. Evans (ME) in Clear Creek County, Colorado, USA. The y-axis represents the autocorrelation function at different lagged values in days (x-axis). The autocorrelation function is calculated between plant productivity values (NDVI) and invertebrate taxa for each year of the study (columns represent specific taxa and rows represent year). The blue dashed lines represent 95% confidence intervals beyond which autocorrelations are statistically different from zero. Negative lag values with positive correlations indicate correlations when invertebrates led NDVI, and positive lag values indicate correlations when NDVI led invertebrates.


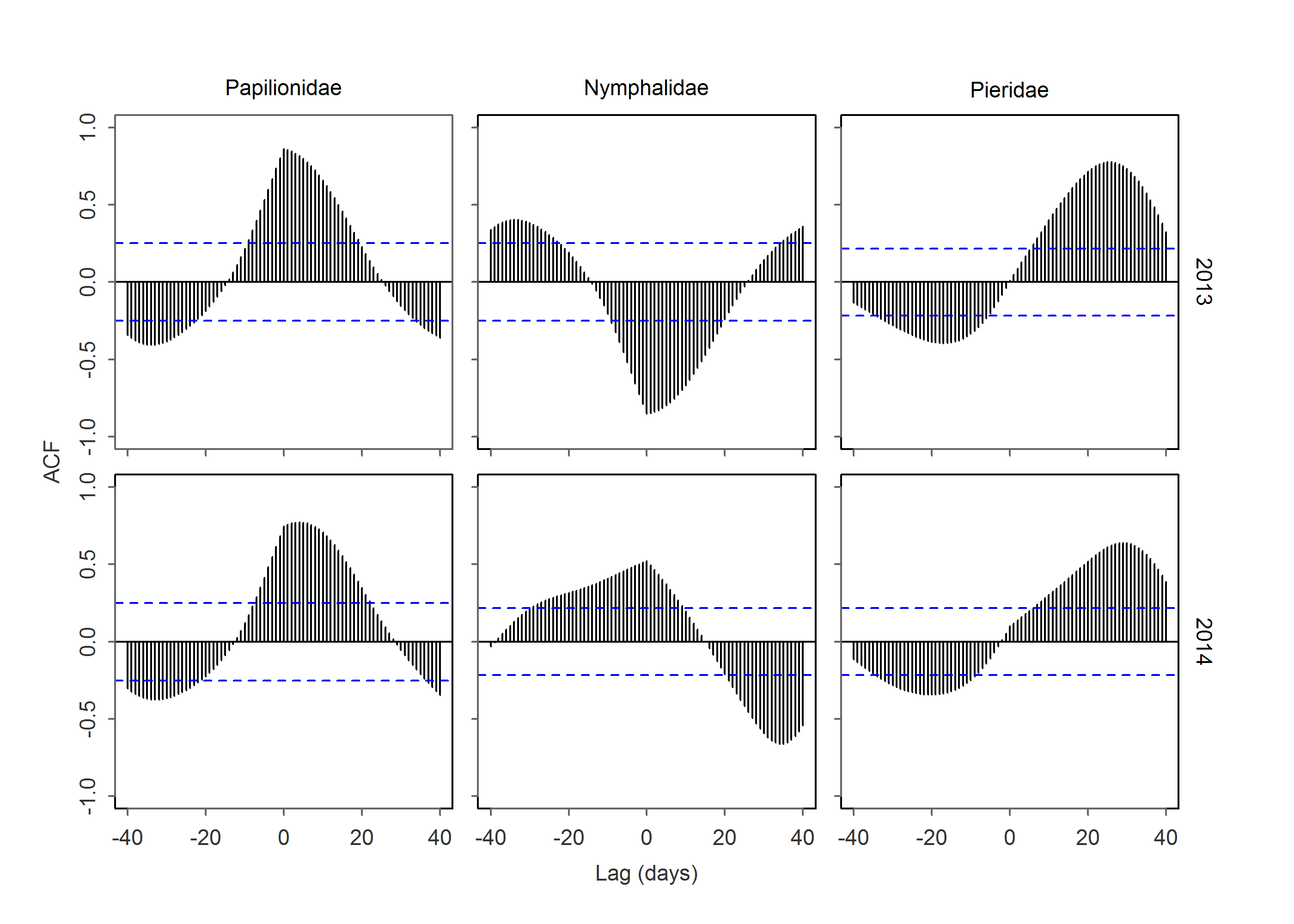


**Figure S8**. Cross-correlations for two temporally-overlapping time series (invertebrate food taxa of unknown forage quality and NDVI) at Mesa Seco (MS) in Hinsdale County, Colorado, USA. The y-axis represents the autocorrelation function at different lagged values in days (x-axis). The autocorrelation function is calculated between plant productivity values (NDVI) and invertebrate taxa for each year of the study (columns represent specific taxa and rows represent year). The blue dashed lines represent 95% confidence intervals beyond which autocorrelations are statistically different from zero. Negative lag values with positive correlations indicate correlations when invertebrates led NDVI, and positive lag values indicate correlations when NDVI led invertebrates.

**
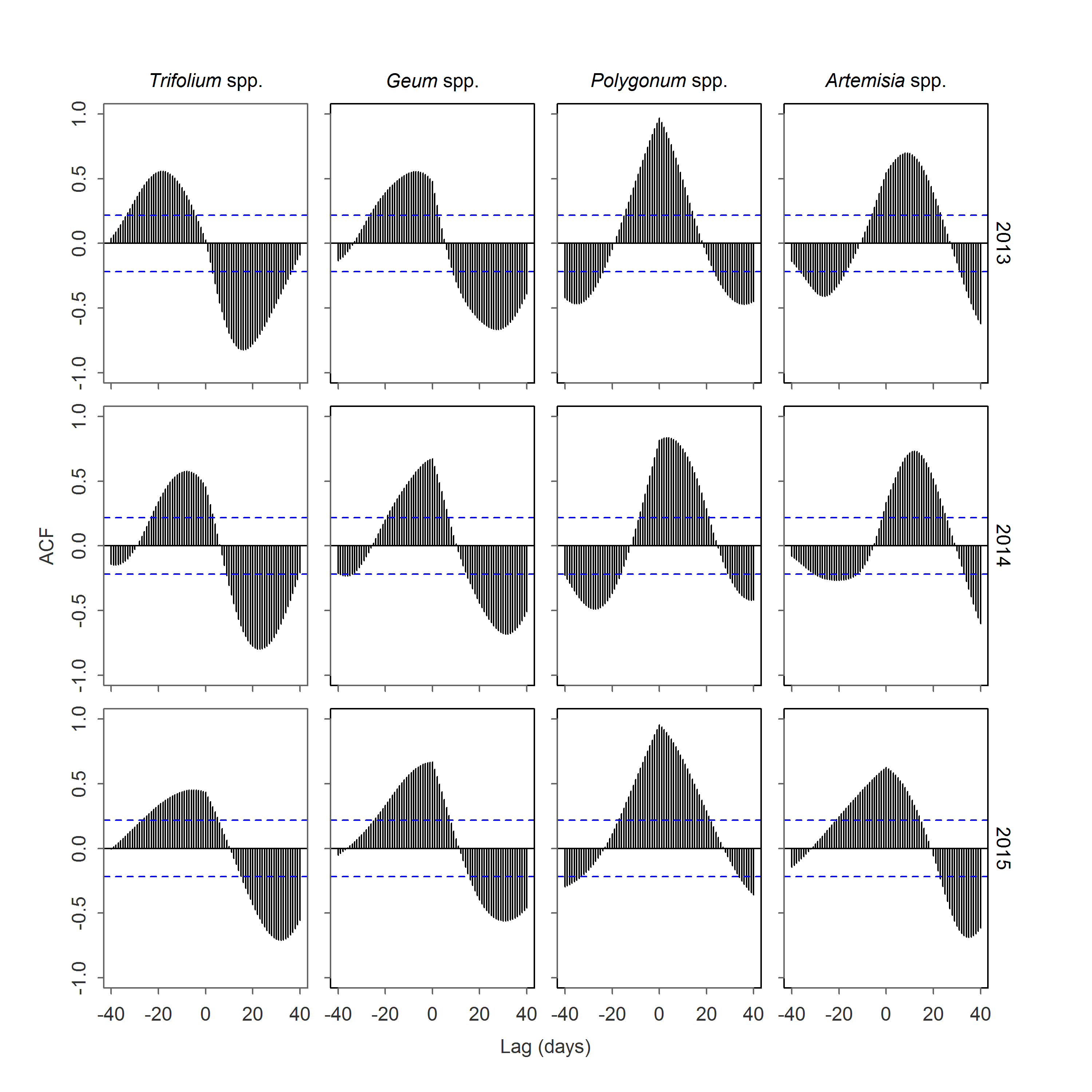
**

**Figure S9**. Cross-correlations for two temporally-overlapping time series (bloom probability of forage species and NDVI) at Mt. Evans (ME) in Clear Creek County, Colorado, USA. The y-axis represents the autocorrelation function at different lagged values in days (x-axis). The autocorrelation function is calculated between plant productivity values (NDVI) and probability of bloom for different forage species for each year of the study (columns represent specific taxa and rows represent year). The blue dashed lines represent 95% confidence intervals beyond which autocorrelations are statistically different from zero. Negative lag values with positive correlations indicate correlations when forage species led NDVI, and positive lag values indicate correlations when NDVI led forage species.


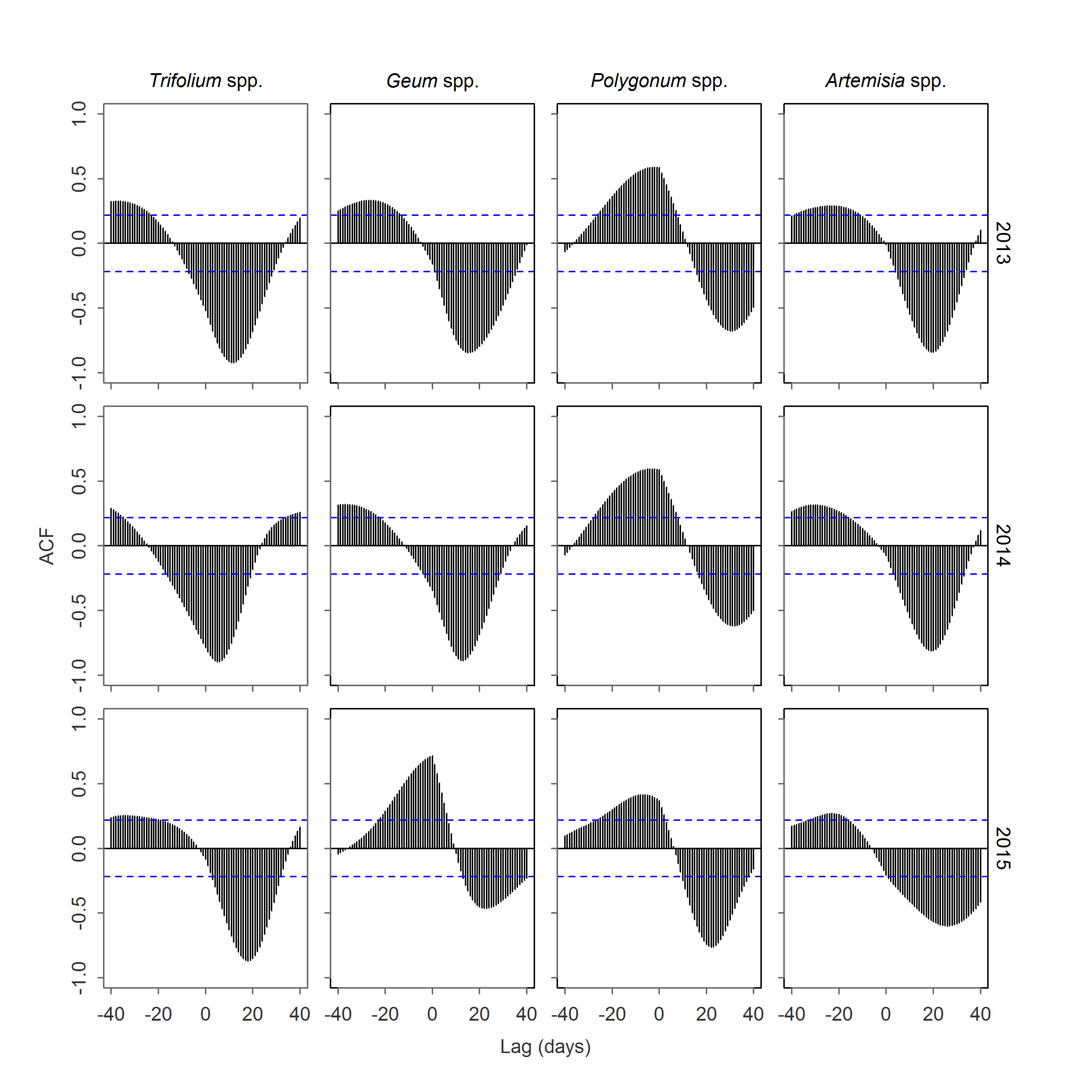


**Figure S10**. Cross-correlations for two temporally-overlapping time series (bloom probability of forage species and NDVI) at Trail Ridge (TR) in Rocky Mountain National Park in Larimer County, Colorado, USA. The y-axis represents the autocorrelation function at different lagged values in days (x-axis). The autocorrelation function is calculated between plant productivity values (NDVI) and probability of bloom for different forage species for each year of the study (columns represent specific taxa and rows represent year). The blue dashed lines represent 95% confidence intervals beyond which autocorrelations are statistically different from zero. Negative lag values with positive correlations indicate correlations when forage species led NDVI, and positive lag values indicate correlations when NDVI led forage species.


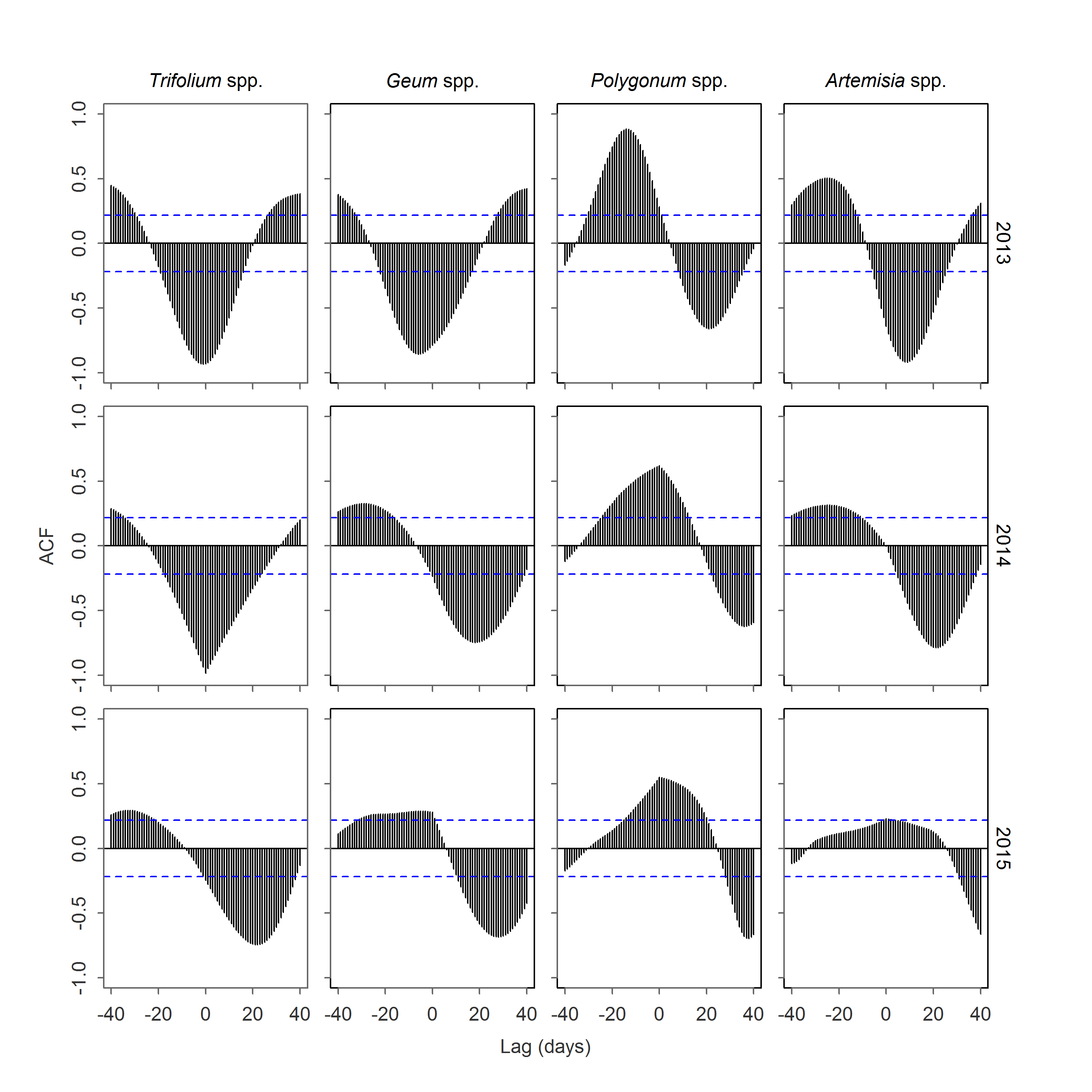


**Figure S11**. Cross-correlations for two temporally-overlapping time series (bloom probability of forage species and NDVI) at Mesa Seco (MS) in Hinsdale County, Colorado, USA. The y-axis represents the autocorrelation function at different lagged values in days (x-axis). The autocorrelation function is calculated between plant productivity values (NDVI) and probability of bloom for different forage species for each year of the study (columns represent specific taxa and rows represent year). The blue dashed lines represent 95% confidence intervals beyond which autocorrelations are statistically different from zero. Negative lag values with positive correlations indicate correlations when forage species led NDVI, and positive lag values indicate correlations when NDVI led forage species.
